# Metabolically regulated spiking could serve neuronal energy homeostasis and protect from reactive oxygen species

**DOI:** 10.1073/pnas.2306525120

**Published:** 2023-11-21

**Authors:** Chaitanya Chintaluri, Tim P. Vogels

**Affiliations:** ^a^Institute of Science and Technology Austria, Klosterneuburg A-3400, Austria; ^b^Centre for Neural Circuits and Behaviour, Department of Physiology, Anatomy and Genetics, University of Oxford, Oxford OX13SR, United Kingdom

**Keywords:** spontaneous activity, mitochondria, reactive oxygen species, metabolism, ion channels

## Abstract

So-called spontaneous activity is a central hallmark of most nervous systems. Such non-causal firing is contrary to the tenet of spikes as a means of communication, and its purpose remains unclear. We propose that self-initiated firing can serve as a release valve to protect neurons from the toxic conditions arising in mitochondria from lower-than-baseline energy consumption. To demonstrate the viability of our hypothesis, we built a set of models that incorporate recent experimental results indicating homeostatic control of metabolic products—Adenosine triphosphate (ATP), adenosine diphosphate (ADP), and reactive oxygen species (ROS)—by changes in firing. We explore the relationship of metabolic cost of spiking with its effect on the temporal patterning of spikes and reproduce experimentally observed changes in intrinsic firing in the fruitfly dorsal fan-shaped body neuron in a model with ROS-modulated potassium channels. We also show that metabolic spiking homeostasis can produce indefinitely sustained avalanche dynamics in cortical circuits. Our theory can account for key features of neuronal activity observed in many studies ranging from ion channel function all the way to resting state dynamics. We finish with a set of experimental predictions that would confirm an integrated, crucial role for metabolically regulated spiking and firmly link metabolic homeostasis and neuronal function.

## Significance

We propose that neuronal action potentials can serve as a protective mechanism to expend surplus energy and maintain metabolic equilibrium, thus preventing toxic conditions in mitochondria. Based on recent experimental findings that link metabolic production and the temporal patterning of neural activity, our work explores potential cellular and biochemical pathways of how extended periods of low energy consumption—e.g., when input activity is low—can lead to the many forms of spontaneous activity that is observed ubiquitously in nearly all nervous systems. We thus provide a fresh look into the complex relationship between neuronal activity and metabolic processes that has direct implications for our understanding of neural communication and could contribute to the development of treatments for neurological diseases.

Neurons fire for no apparent reason (1) in sleeping (2–5), resting (6), and awake animals (2, 7, 8), in acute slices (9–12), cultures (13, 14), and organoids (15). Such spontaneous activity is thought to be an integral part of neural function, but its origin remains unexplained. What’s more, conventional wisdom dictates that spikes relay information based on the inputs they receive. Non-causal activity seems at odds with such a model of efficient neural processing. Finally, spikes are costly, as energy is necessary to power them (16). In this light, it is unclear why neurons should fire in the absence of inputs when it would be less noisy and more energy-efficient to remain passive.

**Fig. 1.**
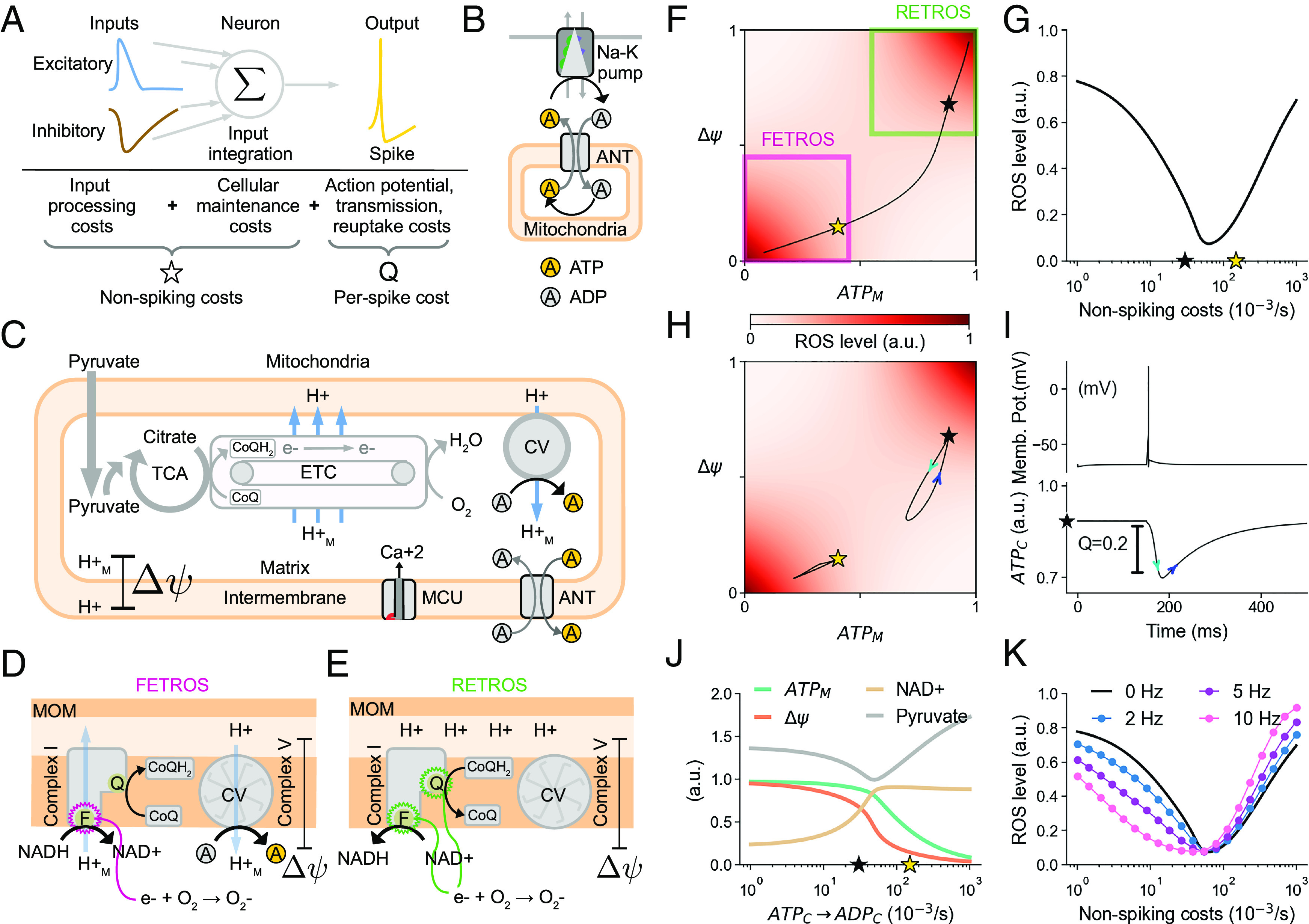
ROS homeostasis by way of metabolic spike generation. (*A*) Schematic of the energy budget of a neuron, including input processing costs (*Left*), cellular maintenance cost (*Middle*), and action potential transmission costs (*Right*). The sum of all input and maintenance costs is indicated by the star symbol and constitutes the non-spiking costs. The additional transient per-spike cost is indicated by Q. (*B*) Metabolic energy is provided intracellularly largely through ATP, which is dephosphorylated into ADP to, e.g., drive ion pumps. This ADP is exchanged with ATP that is replenished in the mitochondria. (*C*) In mitochondria, pyruvate is transformed in the tricarboxylic acid cycle (TCA, or citric acid cycle), ultimately driving protons via the electron transport chain (ETC) into the intermembrane space to generate a gradient (ΔΨ) across the inner membrane. These protons (H+) re-enter the matrix (H+M) through the complex V (CV) and mill ATP from ADP. (*D* and *E*) Reactive oxygen species (ROS) production as a side-effect of proton pumping in the mitochondria: (*D*) “Forward electron transport” ROS (FETROS) is routinely produced during normal function of the electron transport chain when electrons escape, e.g., at complex I during the oxidation of NADH at binding site F. (*E*) “Reverse electron transport” ROS (RETROS) is produced when the electron transport chain is stalled, due to lack of ADP. Stalled electrons escape, e.g., at the ubiquinone-binding site Q. (*F* and *H*) The “respiratory state space” of the extended mitochondrial model is spanned by ATP (x-axis) and ΔΨ (proton gradient, y-axis), which determine ROS production (colormap). The black and gold star mark the corresponding steady state values of ATP${}_{M}$ concentration and ΔΨ for two exemplary metabolic states used throughout this study. (*F*) Areas of FETROS and RETROS production are marked in magenta and green squares, respectively. The black line describes the range of baseline costs. (*G*) ROS levels depend on the baseline (non-spiking) costs and corresponds to the line from *F*. (*H*) Transient trajectory away from the baseline (gold and black stars) as a consequence of a spike. (*I*) Transient decrease in ATP level over time (*Bottom*) as a result of one spike (*Top*). The black star in the *Bottom* plot indicates the baseline cost. Spike-induced excursions in respiratory state space for this spike are shown in *H*. (*J*) The concentrations of mitochondrial ATP (green), NAD+ (tan), ΔΨ (orange) and pyruvate (gray) for different steady states. (*K*) Additional spiking affects ROS levels. ADP: adenosine diphosphate, ATP: adenosine triphosphate, ANT: adenine nucleotide translocator, Ca+2: calcium ions, CoQ/CoQH2: coenzyme Q oxidized/reduced forms, CV: complex V, e-: electron, ETC: electron transport chain, H+: intermembrane proton, H2O: water, MCU: mitochondrial calcium uniporter, MOM: mitochondrial outer membrane, NAD+/NADH: nicotinamide adenine dinucleotide oxidized/reduced forms, O2: oxygen, subscript M: mitochondrial matrix, subscript C: cell cytosol, TCA: tricarboxylic acid cycle.

Energy consumption in neurons, broadly speaking, is the utilisation of ATP for i) restoring ion gradients after synaptic inputs, ii) general maintenance of the cellular machinery, and iii) the generation and transmission of spikes (Fig. 1*A*). Input (i) and output (iii) processing costs are substantial (16, 17), and ATP consumption can fluctuate widely between periods of input silence and intense activity. In order to quickly react to incoming inputs, neurons must near-instantaneously match cellular ATP demands with ATP production. Such rapid ATP production in neurons is facilitated by their mitochondria (Fig. 1*B*), where the chemical energy from pyruvate is transformed in the tricarboxylic acid cycle (TCA, or citric acid cycle) and the electron transport chain (ETC) to establish a proton gradient across the mitochondrial inner membrane denoted by ΔΨ. When protons re-enter the mitochondrial matrix through the complex V (ATP synthase) of the ETC, ATP is milled from ADP molecules (18) (Fig. 1*C*). This mitochondrial ATP is then exchanged with the cytosolic ADP to be consumed in processes such as Na-K pumps.

In all cells—not just neurons—electrons can escape from the mitochondrial ETC during ATP production. These rogue electrons lead to the formation of reactive oxygen species (ROS) which can act as signaling compounds (19), and in higher doses disrupt, and even become toxic (20) for cellular processes (*SI Appendix*). ROS levels increase with increased number of electrons transiting the ETC in a process known as “Forward Electron Transport” ROS (FETROS) release (21). This occurs during periods of increased ATP production that matches high ATP demand. Under these circumstances, any established ΔΨ is used to produce ATP, which in turn is rapidly utilised and turned to ADP. FETROS occurs under high ATP demands and coincides with low ΔΨ and low ATP concentrations (Fig. 1 *D* and *F*, *Lower Left* magenta square).

Critically, decreased energy demands, i.e., lowered ATP consumption, can also increase ROS release (21). This occurs when ATP is not consumed at expected rates and mitochondrial ADP becomes scarce at the complex V. Consequently, ATP production nearly stalls and the electrons lingering on the ETC escape to produce so-called “Reverse Electron Transport” ROS (RETROS) (21, 22). RETROS increases with increased ETC stalling and coincides with high ΔΨ and high ATP concentrations (Fig. 1 *E* and *F*, *Upper Right* green square). Such increased ROS production in case of both increased and decreased ATP production leads to a non-monotonic, V-shaped relationship between ATP consumption and intracellular ROS levels (23) (Fig. 1*G*). ROS is toxic for most cellular processes. For example in skin cells, ROS-induced stress leads to cell death. However terminally differentiated post-mitotic cells, i.e., cells that cannot be replaced or replicated such as skeletal muscle cells, are equipped with additional mechanisms to minimise ROS by scavenging the escaped electrons (24). They may even avoid ROS formation altogether by controlling the intake of glucose—the primary 6-carbon chemical energy supply in cells—and tightly regulating its breakdown to pyruvate via glycolysis, or by storing surplus as reserves in the form of glycogen or lipid droplets.

Neurons are special. Despite their extensive energy demands, healthy, mature, i.e., terminally differentiated and post-mitotic, neurons are not known to maintain long-term energy reserves (25–28). Moreover, glucose intake and its breakdown via glycolysis are less well regulated in neurons (29–34) and continuously augmented by supplements from external sources (28, 35, 36) (For more details on metabolism in neurons, see *SI Appendix*, Figs. S5 and S6, and for metabolic differences between neurons, astrocytes, and skeletal muscle cells see *SI Appendix*, Table S4). These observations lead us to the central assumption of our work—in neurons, ATP production follows a lean production cycle with the rate-limiting step at the last site of the electron transport chain. In other words, the rate of ATP production is controlled by (ΔΨ and) the availability of ADP at ATP synthase (complex V) of the ETC. Functionally, this is sensible because neurons need to retain a high level of metabolic readiness in order to process potentially life-threatening inputs and consequent rapid surges in ATP demands. Keeping the rate-limiting step of ATP production at the complex V can guarantee such readiness. Only a sufficiently high proton gradient and ADP would thus be required to produce ATP. Mechanistically, our assumption is congruent with neurons’ lack of long-chain carbon reserves, their reliance on leaner carbon molecules and on mitochondria for their energy requirements. The brim-full proton gradient that is beneficial to guarantee quick and reliable ATP availability would come at the cost of RETROS-poisoning during periods of synaptic quiescence when ATP production stalls (23). It is conceivable that under RETROS conditions many ATP-consuming processes such as phosphorylation, transport of cellular cargo and cellular upkeep would be enhanced, but given that up to 80% of a neuron’s ATP budget may be allocated to input processing (17), a more comprehensive solution, in addition to ROS scavenging, seems imperative to avoid acute RETROS build-up. (For a list of ROS relieving mechanisms, see *SI Appendix*, Table S3.)

Here, we propose that not all of the ubiquitously observed “spontaneous” firing is in fact not spontaneous, but rather serves as a release valve to protect neurons from toxic reactive oxygen species that appear due to ATP-production stalling when energy consumption is low, i.e., when RETROS conditions prevail. Further, we show that such metabolic spike regulation may be behaviourally relevant and we explore the validity and the implications of our theory in 5 increasingly detailed, biologically plausible models. We begin with a mitochondrial model that links the TCA and ATP production with ROS release to show that I) metabolic spiking, i.e., spiking that serves metabolic homeostasis and save neurons from ROS, could provide the necessary quick increase in ATP expenditure and limit RETROS. Next, II) we account for the frequency and the firing pattern of metabolic spiking that would be necessary to perform ROS homeostasis and explore potential mechanisms that can detect a neuron’s metabolic state and drive the general changes in intrinsic firing. To showcase a single ion channel type’s response to ROS more concretely, III) we model the reported intrinsic firing changes in dorsal fan-shaped body neurons due to β-subunits of Shaker channel of fruit fly (*Drosophila melanogaster*). Further, we show that metabolically regulated spiking may not just contribute noise, but may be integral for neural function and behaviour. For this, we examine IV) the consequence of metabolically regulated spiking in a large-scale recurrent network model and compare the resulting sustained activity with previous experimental observations. Finally, we end our study with a set of experimentally testable predictions V) that would prove or falsify our theory.

## Theory

Formally, we state our theory as follows:

Assumptions—i) In neurons, the rate-limiting step of ATP production is located at its last step, i.e., complex V (ATP-synthase) of the electron transport chain in their mitochondria. ii) Neurons, depending on their physiological operational conditions, can face a range of metabolic demands and consequently may be exposed to non-monotonically varying ROS levels. At ideal metabolic preparedness, ROS levels are minimal and increase both when higher (FETROS) and lower (RETROS) energy demand occurs.

Hypothesis—Neurons sense their metabolic state and modulate their excitability to achieve metabolic homeostasis, thus minimizing ROS. Toward this goal, neurons may initiate metabolic spiking under RETROS conditions and limit their spiking during FETROS conditions.

## Results

In neurons, several cellular processes consume ATP. In our study, we separated all ATP-consuming processes into two categories based on how quickly these ATP costs can change. First, we consider the ATP budget for all input processing and cellular maintenance as a slow-changing, steady-state expense. We refer to this budget as the non-spiking cost or baseline cost and mark it as a star (⋆) in all figures. At baseline, the ATP-producing mitochondrial processes will match the ATP demands and arrive at steady state values of ATP concentration and ΔΨ (Fig. 1*A*). Second, we consider the ATP budget that is required to re-balance the Na-K ions, neurotransmitter release, and re-uptake after an action potential as a fast transient increase from the baseline costs. We denote this budget as per-spike cost (Q) (Fig. 1 *A* and *I*).

To visualize the baseline costs and the transient excursions due to a spike, we introduce the ‘respiratory state space’ (Fig. 1 *F* and *H*)—a two-dimensional space spanned by ATP on the x-axis and ΔΨ on the y-axis. Any given neuron will be situated at a specific location in respiratory state space according to its current baseline cost (Fig. 1*F*). Spikes will produce transient excursions along trajectories that depend on a neuron’s position in the state space and the per-spike cost expense Q. The neuron’s momentary position thus reflects its metabolic state.

### I. Metabolic Spiking Can Quench Mitochondrial ROS.

Periods of low ATP consumption are known to lead to elevated ROS levels in other cell types (21), but they have never been observed in neurons, despite their widely fluctuating energy consumption. We wondered whether spike initiation could serve as a homeostatic mechanism that regulates ROS by restoring ATP production when energy consumption is low. To explore this idea more quantitatively, we developed a theoretical framework in which the metabolic state affects neural excitability. We started by expanding an existing mitochondria model (37) to include ROS production and its scavenging such that ROS amplitude was a direct consequence of the concentration of mitochondrial ATP (ATP${}_{M}$) and the proton gradient ΔΨ (Fig. 1*F*–*H* and *J* and Materials and Methods).

ROS concentrations could thus be regulated by eliciting “metabolic spiking,” prompting excursions from their momentary metabolic state (black star in all figures) in the “respiratory state space” (Fig. 1 *F* and *H* and *SI Appendix*, S1 *B* and *C*). These excursions (Fig. 1 *H* and *I*) from non-spiking baseline transiently decrease cytosolic ATP (ATP${}_{C}$) and thus provide mitochondrial ADP (ADP${}_{M}$). Depending on a neuron’s position in respiratory state space, additional spikes can decrease or increase ROS (Fig. 1*H*, black and gold star, respectively) independently of other variables (*SI Appendix*, Figs. S1 and S2). To minimise FETROS, a neuron may thus have to decrease spiking, and save ATP. To minimise RETROS, a neuron may instead have to produce spikes at various intervals and thus spend ATP (Fig. 1*K*). Because ROS production is non-monotonic with ATP consumption, there exists an ATP expenditure rate $\bar{\;\text{ATP}}$ between the RETROS and FETROS conditions such that ROS levels are at a minimum (Fig. 1*G*).

### II. The Firing Patterns a Neuron Produces Will Depend on the Details of Its Energy Budget.

**Fig. 2.**
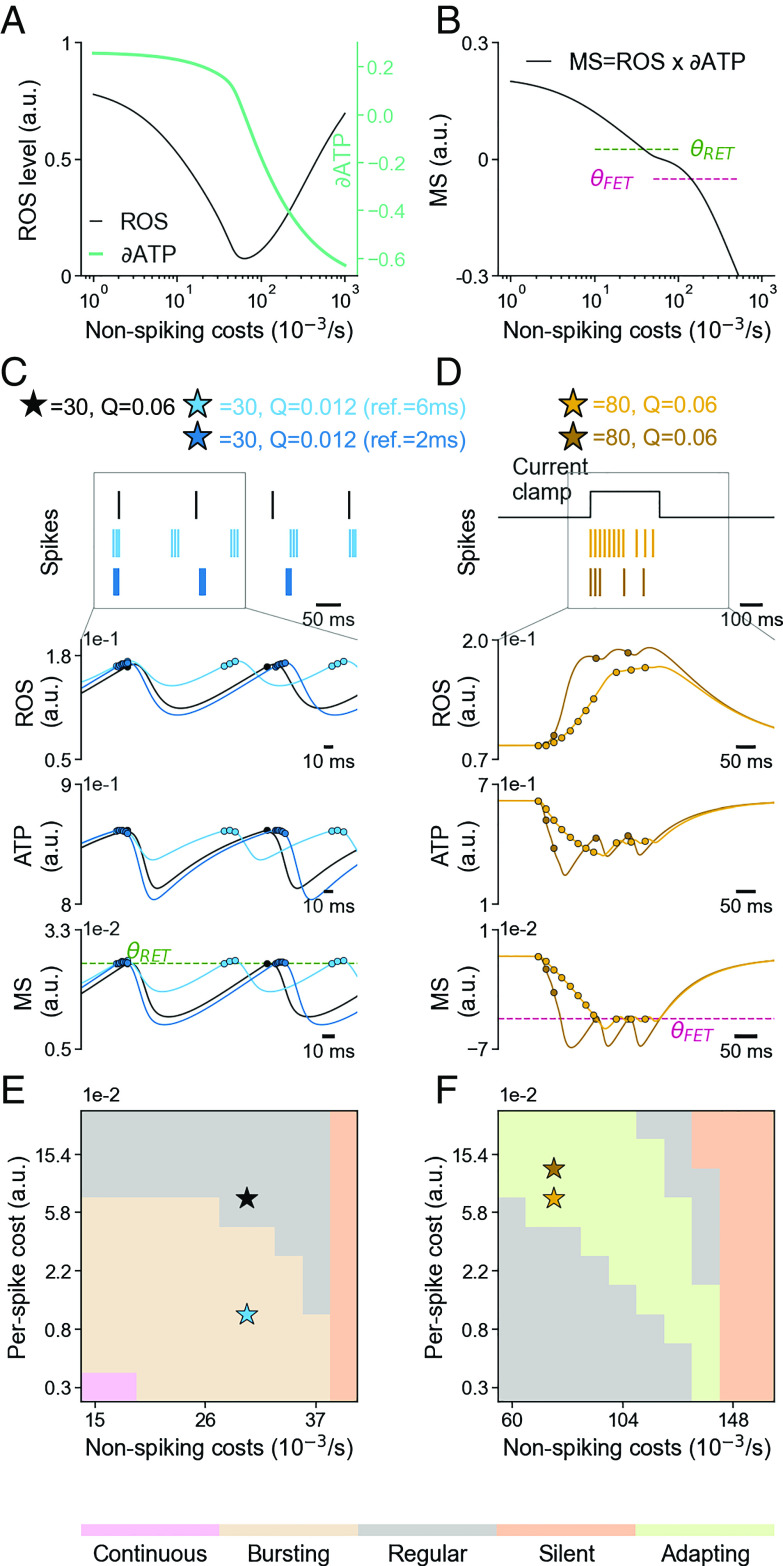
ROS homeostasis is linked to neural excitability. (*A*) ROS and ∂ATP levels change with non-spike related cytosolic ATP${}_{C}$ consumption in our simplified metabolic accounting model. (*B*) The metabolic signal (MS) as the product of ROS and ∂ATP. Two thresholds $\theta_{\mathrm{RET}}$ (dotted green line) and $\theta_{\mathrm{FET}}$ (dotted magenta line) control the neural response. (*C*) *Top Panel*: Raster of metabolic spikes initiated when the MS $>\theta_{\mathrm{RET}}$ (black). When Q is low, MS remains higher than $\theta_{\mathrm{RET}}$ after the refractory period and additional metabolic spiking is initiated (light blue). Lowering the refractory period increases in-burst frequency and decreases inter-burst frequency (dark blue). The *Lower* panels show the ROS, ATP, and MS levels for 120 ms of the simulation (second, third, and fourth row, respectively) for the three cases. Spikes are marked as “o.” (*D*) *Top Panel*: Raster of spikes (gold) in response to an external current source (black). When Q is high such that MS decreases and stays $<\theta_{\mathrm{FET}}$ for longer (brown) where spiking is prohibited. The *Lower* panels show the ROS, ATP, and MS level (second, third, and fourth rows). Spikes are marked as “o.” (*E* and *F*) The non-spiking related baseline ATP usage (x-axis) and the per-spike-cost Q (y-axis) determine the spectrum of intrinsic firing responses ranging from silent to continuously spiking neurons in RETROS (*E*) and FETROS (*F*) conditions.

In a minimal neuron model that tracks the metabolic costs of basic maintenance, integration, and spiking (Fig. 2*A*) we can estimate the firing patterns a neuron must elicit to reach a ROS minimum.

In a real neuron, the metabolic state could be read out from its ROS levels directly (see below), but also from its ATP/ADP ratio, its intracellular Ca2+ concentration, and the cumulative footprint of its ROS scavenger response with its resulting redox potential. For the simplicity of our model, we considered only two of these variables and calculated a non-ambiguous “metabolic signal” as MS = ROS ×∂ATP, where ∂ATP = ATP −$\bar{\;\text{ATP}}$ (Fig. 2*B*).

In this accounting model (*SI Appendix*), the necessary spike changes to minimize ROS can be obtained by introducing two thresholds, $\theta_{\mathrm{RET}}$ and $\theta_{\mathrm{FET}}$. When MS is large (high ROS, high mitochondrial ATP${}_{M}$), such that MS $>\theta_{\mathrm{RET}}$, the model elicits ATP-consuming, RETROS-quenching metabolic spiking (Fig. 2*C*). If MS decreases, such that $\theta_{\mathrm{RET}}>$ MS $>\theta_{\mathrm{FET}}$, metabolically regulated spiking ceases. When MS decreases further (depleted mitochondrial ATP${}_{M}$, high ROS), such that MS $<\theta_{\mathrm{FET}}$, even input-driven, synaptic spiking is prevented (Fig. 2*D*). In our model, intrinsic excitability is thus governed by MS and serves as a homeostatic mechanism to minimize ROS.

The effectiveness of a spike to modulate ROS depends on a number of variables, most prominently the transient increase in ATP consumption after each spike, i.e., per-spike-cost (Q) (Fig. 1*A*). When the per-spike-cost Q is sufficiently high, single spikes delivered at low rates suffice to quench RETROS. For lower Q, several spikes are necessary to decrease the MS and quench high ROS (Fig. 2*C*). Q also affects the FETROS response, as expensive spikes will deplete cellular ATP more and lead to prolonged periods of blocked spike initiation (Fig. 2*D*). The various possible combinations of Q and steady-state ATP consumption can account for many different spike patterns, ranging from silent to regular firing to bursting, to continuously active (Fig. 2 *E* and *F*). In mature neurons, Q would presumably be constant, but the steady state non-spiking expense (star in all figures) may change, e.g., due to changes in input activity, a neuron’s size or its history (see *SI Appendix*, Fig. S3 for additional relevant variables). The firing patterns that emerge from these combinations of constraints can range from an occasional single spike to bursting or even continuous spiking and mirror many of the experimentally observed intrinsic activity patterns (*Discussion*).

**Fig. 3.**
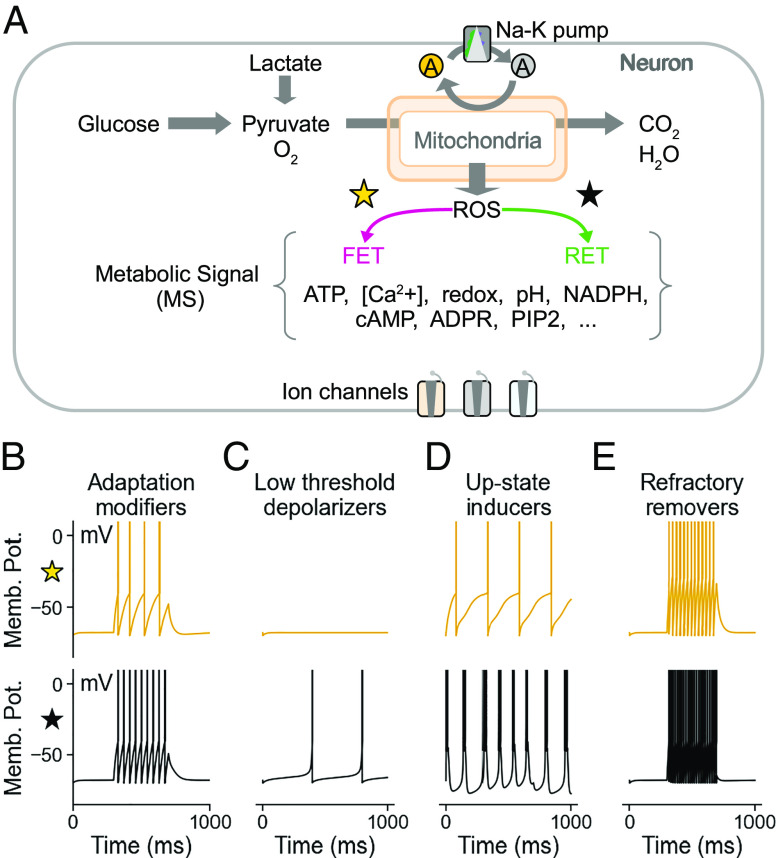
ROS homeostasis could be mediated by ion channels. (*A*) ROS, its scavenging response by the cell, concentration of various substrates, its redox state, Ca2+ concentration, pH, etc, determine the cellular metabolic state (position of the star in Fig. 1*J*) and cause changes in ion channels or their sub-units to modulate neuronal excitability. (*B*–*E*) Schematic of four discernible firing pattern changes mediated by ion channel modifications. (*B*) Increased participation of A-type channels in spike repolarization can facilitate high frequency firing by diminishing spike adaptation. (*C*) Opening of low threshold depolarising channels (e.g., persistent Na or TRPM2 channels) and the closure of hyperpolarizing channels (e.g., ATP-dependent K channels and SK channels) can cause metabolic spiking. (*D*) Increased conductivity of T-type Ca2+ channels can promote bursting e.g., by limiting Na channel inactivation. (*E*) Modification of ion channels sub-units can eliminate refractory period between spiking (e.g., seen as a consequence of resurgent Na currents).

Any energy homeostatic mechanism must be sensitive to the current metabolic state of the neuron. We propose that excitability of the neuron must dynamically change in response to the metabolic status, and provide relief from both FETROS and RETROS. A candidate mechanism to achieve such a swift modulation in excitability would likely involve the many ion channel types that can sense the metabolic state via metabolic signals (Fig. 3*A*). We can broadly categorise such metabolic state-sensing ion channels as ATP “savers” and “spenders” in a simplified neuron model (*SI Appendix*).

ATP savers aim to silence neurons when FETROS conditions prevail. Their combined action delays spikes or suppresses them entirely. ATP saver channels act to hyperpolarize the membrane potential such as ATP-sensitive or Ca2+-sensitive potassium channels (Fig. 3*B*, *Top*). On the other hand, ATP spenders should facilitate spiking, e.g., through channels that aid rapid re-polarisation and decrease spike-frequency adaptation (Fig. 3*B*, *Bottom*) or by low-threshold-activating depolarising channels (Fig. 3*C*). Similarly, channels that would increase bursting (Fig. 3*D*), or shorten the refractory period and thus effectively eliminate interspike intervals would promote ATP spending and support the firing patterns required to maintain a baseline level of ATP expenditure (Figs. 2*E* light purple and 3*E*).

Cumulative changes in many ion channels, such as changes in voltage sensitivity, activation or inactivation profiles, time constants and conductivity, and several other post-translational modifications in response to the metabolic state, will determine whether a neuron decreases (Fig. 3*B*–*E*, *Upper Panels*) or increases (Fig. 3*B*–*E*, *Lower Panels*) its excitability (*Discussion*). Importantly, the down-regulation of ATP savers during RETROS could effectively permit ATP expenditure, and vice versa. As such, the designation of an ion channel as an ATP saver or spender would not be based on the specific ion they gate, but on the cumulative change in the firing property of the neuron. Notably, we can identify more than 15 candidate pathways (*SI Appendix*, Table S2) that target ion channels which could counter RETROS or FETROS conditions.

### III. Linking Ion Channel Function with Metabolic State and ROS.

**Fig. 4.**
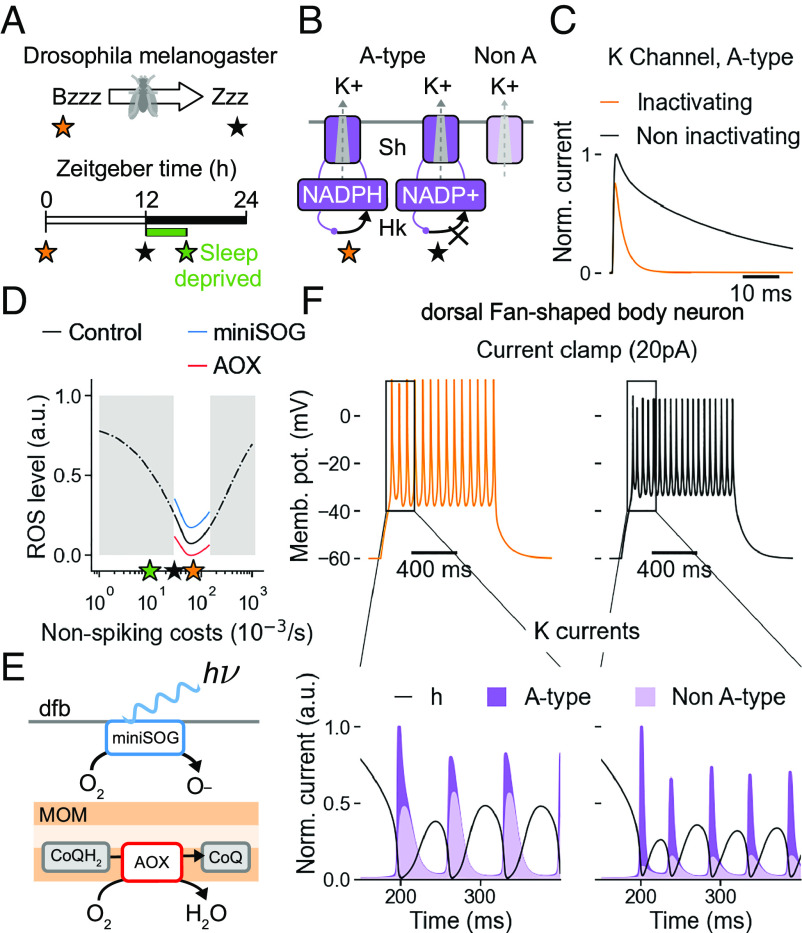
Functional consequences of ROS homeostasis. (*A*) Changes in intrinsic firing of the dorsal fan-shaped body neuron in fruit flies may be related to changes from high (orange star) to low metabolic state (black star) and switch the behavioural state from awake to sleep (38). (*B*) The origin of firing changes is thought to lie in the altered inactivation mechanism of NADPH-receptive Hyperkinetic β-subunits (Hk) of Shaker (Sh) channels. Changing their profile from inactivating (orange star) to non-inactivating (black star) affects the ratio of A (dark purple) to non-A (light purple) type potassium currents. (*C*) Voltage clamp response of inactivating (orange) and non-inactivating (black) A-type currents for a 40-mV step. (*D*) Changes in ROS production (black line) as a function of non-spiking costs. The neurons’ presumed physiological range is indicated in white background. Sleep-deprived state with its hallmark of high firing and increased ROS levels lies outside of the physiological range (green star) (38). ROS production can be altered by way of miniSOG (blue) and AOX (red). (*E*) miniSOG increases ROS optogenetically (*Top*); overexpressing AOX oxidizes coenzyme-Q and decreases ROS levels (*Bottom*). (*F*, *Top*) Spiking response to a 20 pA current injection in neurons with inactivating (orange) and non-inactivating (black) K current; *Bottom*) zoomed-in visualization of the inactivating (*Left*), or non-inactivating (*Right*) A-type (dark purple) and non-A-type (light purple) K current contributions, co-plotted with the sodium current’s inactivation variable h (black).

One example of using spikes to sustain the ETC has been observed in dorsal fan-shaped body (dfb) neurons of *Drosophila melanogaster* (38) (Fig. 4*A*). In these sleep-switching dfb neurons, the A-type “Shaker” potassium channels’ β-subunits called “Hyperkinetic” are modulated by a key molecule involved in ROS scavenging called nicotinamide adenine dinucleotide phosphate (NADPH) (Fig. 4*B*). When ROS is high, NADPH is converted into NADP+, neutralising ROS, but also eliminating the channels’ inactivation mechanism. The fast-activating, fast-inactivating Shaker-Hyperkinetic channel thus changes to slow-inactivating during RETROS states (Fig. 4*C*). This loss of the inactivation gate of a K+ channel is thought to be the cause for a behaviourally relevant increase in firing (Fig. 4*F*, *Top*).

We can support this assumption and reproduce ROS-dependent firing changes in a standard Hodgkin–Huxley type model with an additional A-type K+ channel, resembling Shaker-Hyperkinetic channel, that alters its inactivation properties in response to ROS. In our model (*SI Appendix*), slowed inactivation of the A-type K+ channel leads to increased participation of its currents in membrane re-polarisation, out-competing slower-activating, non-A-type currents and thus allowing for more rapid re-polarisation (Fig. 4*F*, *Bottom*) and consequently decreased inter-spike intervals (and thus an increase in firing) as consistent with experimental observations.

In the experiment, this can be directly linked to ROS because, when the fruit fly is sleep deprived (Fig. 4*D*, green star), the dfb neurons are pushed beyond their physiological operational preparedness (Fig.4*D*, gray background). In our framework, this can be interpreted as a situation in which metabolic homeostasis through spiking becomes insufficient and the additional RETROS burden is revealed. In the experiment, a higher ROS burden can also be delivered optogenetically through mini Singlet Oxygen Generators (miniSOG) and this can be simulated congruently by an upward shift in the relationship between baseline ATP consumption and ROS concentrations (Fig. 4 *D* and *E* blue). Conversely, a ROS decrease can be achieved experimentally by Alternative Oxidase (AOX), providing an additional exit for electrons from the ETC (Fig. 4 *D* and *E* red). In the model this can be simulated accordingly by a downward shift in the relationship between baseline ATP consumption and ROS concentrations (Fig. 4*D*). We wondered whether such consistency between model and experiment could also be observed in other neural systems.

### IV. Metabolic Spiking in a Recurrent Neural Network Model.

**Fig. 5.**
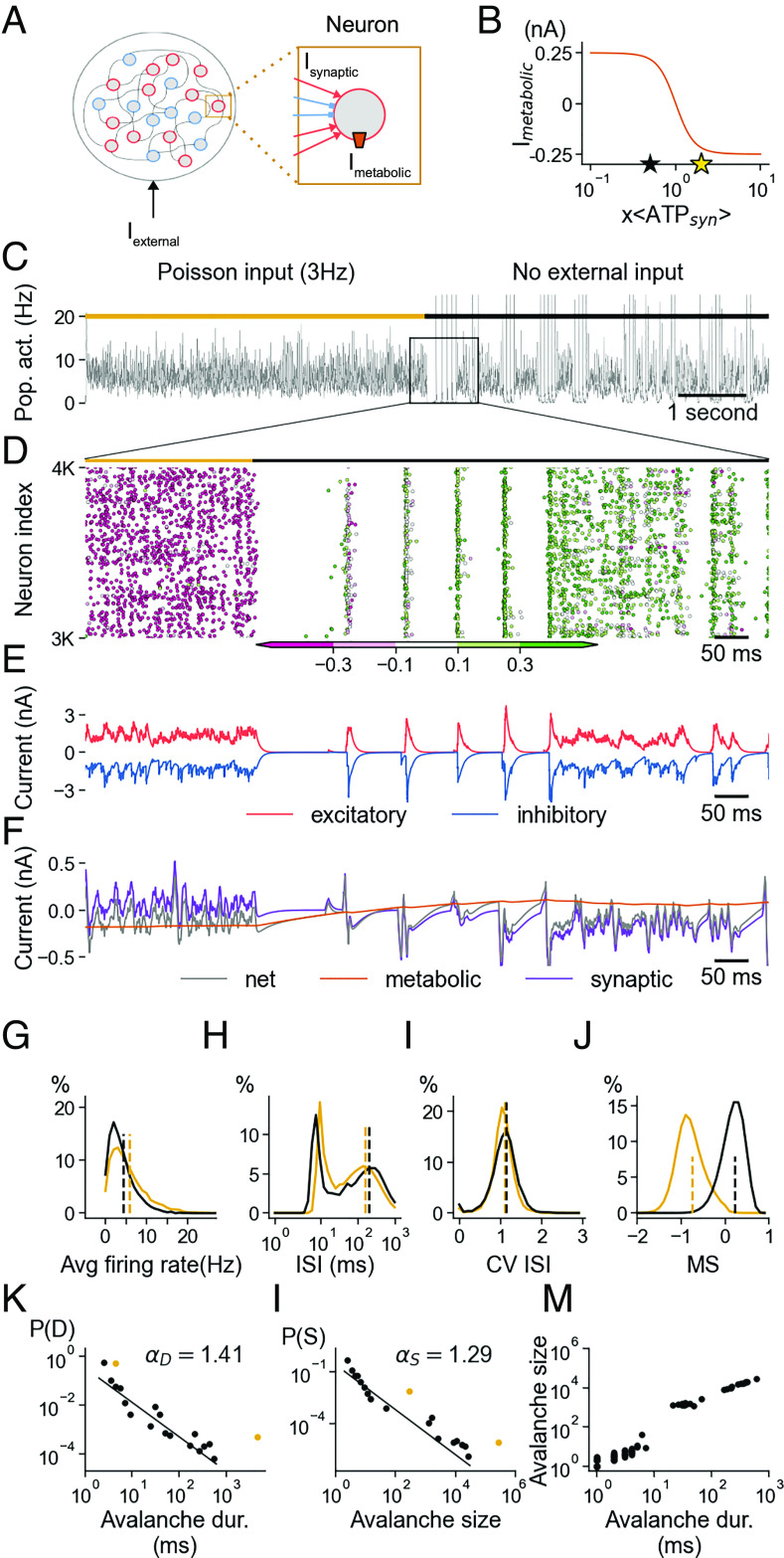
Functional consequences of metabolic spiking in a recurrent network. (*A*) Schematic of a recurrent network model with excitatory (red) and inhibitory (blue) neurons that are equipped with a metabolic spiking mechanism (orange box in the single-cell *Inset*, *Middle*). (*B*) I${}_{\;\text{metabolic}}$ contributes a homeostatic current according to a neuron’s energy consumption due to synaptic currents I${}_{\;\text{synaptic}}$. (*C*) Network population rate over 10s in 1ms bins, with (gold bar) and without (black bar) external Poisson input. (*D*) Spike raster inset of 1,000 randomly chosen neurons during the transition from externally driven spiking to internally generated activity. The colour of each spike reflects the metabolic state of its neuron, ranging from ATP-depleted (magenta) to ATP-rich (green). (*E*) Excitatory (red) and inhibitory (blue) synaptic currents of an example neuron during the transition. (*F*) 5-ms moving average total synaptic current (purple), metabolic current (orange), and net sum of all currents (gray) during the transition for the neuron in *E*. (*G*–*J*) Distributions of neuronal firing rates (*G*), their interspike intervals [ISI, (*H*)] coefficient of variation (CV) of ISIs (*I*), and metabolic signal at time of spiking (*J*) before (gold) and after (black) the transition. The dashed lines show the mean of each distribution. Distribution of duration (*K*) and size (*L*) of continuous activity (separated by at least 1 ms of silence) in the network before (gold) and after (black) the transition. Poisson-driven activity remains “on” forever. The duration of metabolic “avalanches” follows a power law distribution (black line). (*M*) The avalanche size and its corresponding duration are also shown.

To explore the effects of spike-induced energy homeostasis in neural circuits, we built a network model (39) of 8,000 excitatory and 2,000 inhibitory conductance-based leaky integrate-and-fire neurons. We used previously published neuron and network parameters, such that each neuron received recurrent inputs from a random 2% of the network as well as from external Poisson inputs. Additionally, each neuron is subject to a metabolic current $I_{M}$ that was controlled by a metabolic state variable MS (Fig. 5*A* and *SI Appendix*) tracking the metabolic costs of synaptic inputs and action potentials. MS controlled the metabolic current $I_{M}$ such that high MS produced depolarising membrane currents, and low MS lead to hyperpolarising currents (Fig. 5*B*). When the network receives external stimuli, it initially fired at high rates and thus utilised large amounts of ATP, consequently operating in the FETROS regime with very low MS values. As a result, the MS-induced metabolic current becomes hyperpolarizing, contributing to a stable balance of excitation and inhibition and lowering firing rates to prevent seizure-like states (Fig. 5*C*–*F*, *Left*). Such dampened activity shifts the metabolic load near the ROS minimum. When external inputs are turned off, recurrent synaptic activity alone cannot self-sustain indefinitely, and network activity collapses (Fig. 5 *C* and *D*, end of the yellow bar). Due to the lack of synaptic input, and subsequent decrease of ATP consumption, the MS-controlled current becomes depolarising, eventually causing metabolic spiking (Fig. 5*C*–*F*, *Middle*). These first few spikes in the network rapidly activate their similarly depolarised downstream neighbors, and eventually activate the entire network, preventing RETROS build-up via synaptic currents and spiking (Fig. 5*C* –*F*, *Right*). Depending on various variables such as per-spike cost, as well as the structure of the network, activity may briefly cease again (Fig. 5*C*–*F*, *Right*), before experiencing the next RETROS induced avalanche (12).

Such avalanches are indistinguishable from externally driven activity (Fig. 5*G*–*I*), except for their internal metabolic status (Fig. 5*J*). The duration of the activity exhibits a power-law distribution, similarly to widely observed spontaneous activity in experimental set-ups (12, 40) (Fig. 5*K*–*M*). Despite the absence of external inputs, the network responded with stable and robust activity for a wide range of synaptic parameters (*SI Appendix*, Fig. S4). The range of synaptic strength values for which the network displays asynchronous and irregular activity is substantially bigger than reported in previous studies (39) (Fig. 5*C* –*F*, *Left* and *SI Appendix*, Fig. S4 *A*–*C*). In fact, as long as the metabolic currents were not maxed out (and overwhelmed by synaptic currents, *SI Appendix*, Fig. S4*G*), MS allowed the network to self-tune its population rate (*SI Appendix*, Fig. S4*A*) and asynchronicity (*SI Appendix*, Fig. S4 *C* and *E*) to remain persistently and indefinitely active even for parameters settings that would otherwise lead to severe instabilities (*SI Appendix*, Fig. S4 *B*, *D*, *F*, and *J*). Beyond the implication in avalanche-like activity in neuronal networks (Fig. 5*G*–*M*), metabolic regulation of spiking can thus provide sustained, network-wide, asynchronous irregular activity that is thought to be the basis of robust neural circuits (41).

### V. Testable Predictions.

**Fig. 6.**
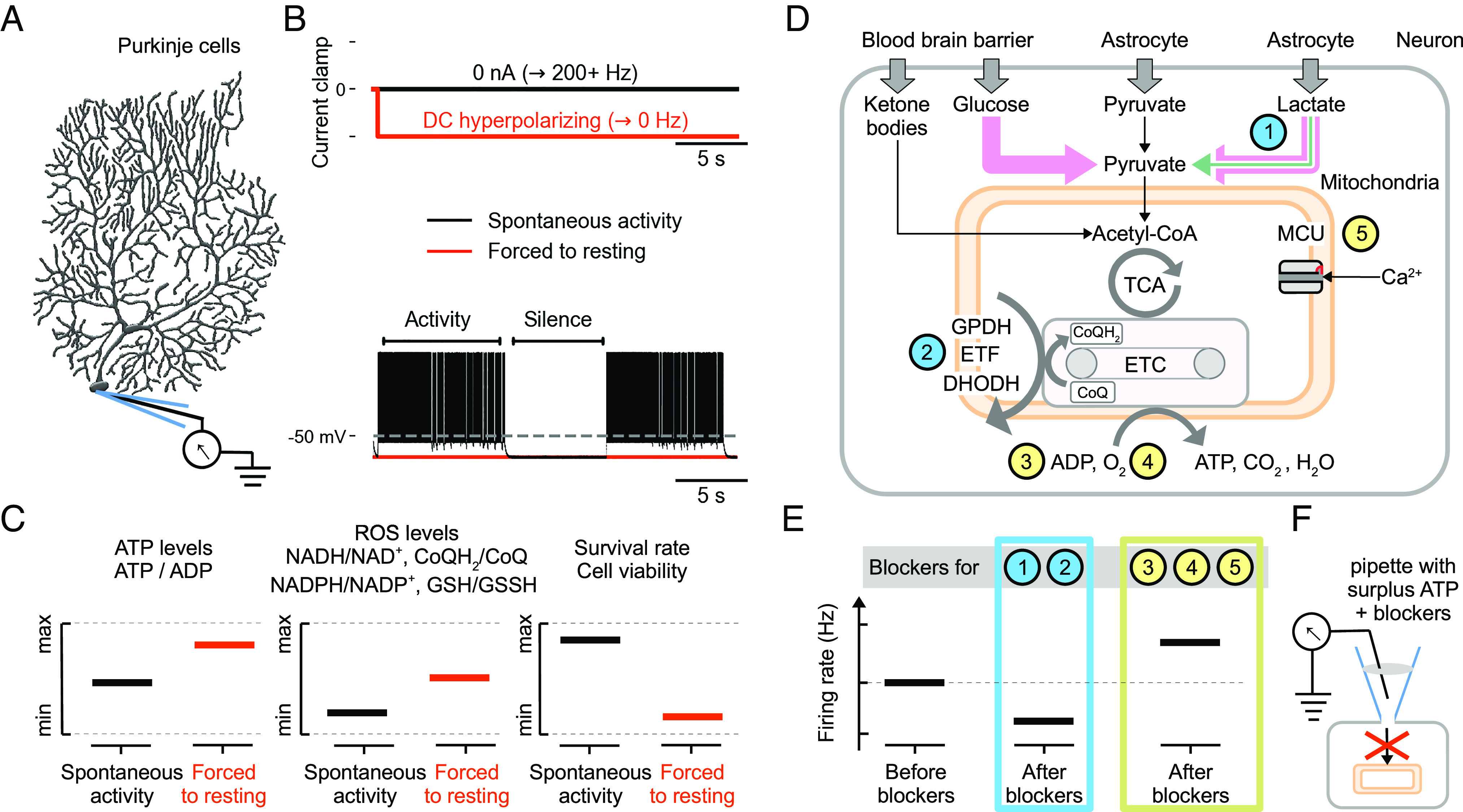
Predictions to test the metabolic excitability hypothesis. (*A*) Metabolic spiking may play a critical role in the cell survival especially in neurons naturally exhibiting spontaneous activity such as Purkinje cells. We predict that (*B*) forcing such spontaneously active cells to resting by DC hyperpolarizing currents would lead to (*C*) increased intracellular ATP levels and a subsequent increase (RET) ROS levels. This perturbation in the long term would decrease the survival rate of neurons. We also predict that the metabolic spiking is initiated due to ATP production stalling in neuronal mitochondria, and to test this, we propose experiments which decouple mitochondria from the neuron at select junctions (*D* and *E*). (*D*) Schematic of the metabolic relationships in neurons, with numbered locii of possible intervention. Neurons (gray box) rely mostly on glucose under FETROS (left pink arrow) and lactate during RETROS and FETROS (right green+pink arrow) for their pyruvate supply. Pyruvate is processed in the neuronal mitochondria (peach box) in their tricarboxylic acid cycle (TCA) and subsequently in the electron transport chain (ETC) to produce ATP and H2O by consuming ADP and O2. Other metabolites such as ketone bodies from blood directly enter TCA via Acetyl-CoA and circumvent pyruvate. While maintaining ample ATP in the pipette, (*E*) blocking the numbered pathways (1,2) in neurons that can limit RETROS conditions may lead to a decrease in the spontaneous firing rates (blue). Conversely, blocking pathways (3 to 5) that would simultaneously increase the ETC stalling and ΔΨ can cause membrane potential depolarization or an increase in the firing rates (yellow). See text for more specific details. (*F*) These interventions must be conducted with ample ATP supply to promote RETROS during testing.

We have argued that neurons experience ROS and in particular RETROS as a result of a high proton gradient in the absence of mitochondrial ADP. Spiking can serve as a means to resupply ADP to a stalled ETC and thus maintain minimal RETROS levels. Our theory is based on a considerable body of indirect evidence (*SI Appendix*, Table S1), but because ROS is detrimental to a cell’s health, we can make a number of experimental predictions to test our theory directly. For example, Purkinje neurons are known to exhibit so-called “simple spikes” spontaneously (Fig. 6 *A* and *B*), in the light of our framework potentially as a homeostatic response to minimize RETROS. By forcefully silencing Purkinje cells with a DC hyperpolarizing current (Fig. 6*B*), we would suppress such ongoing spikes. Following our theory, such a perturbation should result in increased ATP levels (Fig. 6*C*), increased ROS (RETROS) and accelerated cell death, confirming the survival-critical nature of these spikes.

To validate that some “spontaneous” spikes can be of metabolic origin, we propose to decouple metabolism and its hypothesised neural response during periods when the neuron is compensating for RETROS. For instance, under ATP-surplus cytosolic conditions, interventions that decrease the number of stalled electrons on the ETC, or more precisely interventions that decrease the pool of electron-carrying coenzyme-Q molecules (CoQH2) on the ETC (Fig. 6*D*, 1 to 2), such as

1. blocking lactate dehydrogenase (LDH1/LDHB) that would decrease pyruvate production and would slow the tricarboxylic acid cycle (TCA), or
2. blocking pathways that are independent of TCA, that also transfer electrons into the ETC, such as mitochondrial glycerol-3-phosphate dehydrogenase (GPDH), electron-transferring flavoprotein complex (ETF), and dihydroorotate dehydrogenase (DHODH),

should result in decreased metabolic firing rates and generally lowered neuronal excitability. According to our framework, the above perturbations (1 to 2) should result in a decreased RETROS production and thus decreased numbers of metabolic spiking for the same homeostatic effect.

We can also make a number of predictions for perturbations downstream from the ETC that increase the number of stalled electrons (Fig. 6*D*, 3 to 5). More precisely, for interventions which increase the pool of coenzyme-Q molecules carrying these electrons on the ETC (CoQH2), we expect under ATP-surplus cytosolic conditions that:

1. blocking the exchange of cytosolic ADP with mitochondrial ATP at the adenine nucleotide translocator (ANT),
2. limiting O2—which is the usual safe exit of the electrons on the ETC (complex IV)—while maintaining the cytosolic Na+ concentrations, or
3. limiting RETROS relief per-spike by blocking the mitochondrial Ca2+ uniporter (MCU mode 2, *Discussion*),

should lead to further stalling of the ETC and increased ΔΨ across the mitochondrial intermembrane space, and therefore increase RETROS. Under these perturbations, neurons should thus modulate their excitability to increase the metabolic firing.

## Discussion

In cardiac mitochondria, it has been shown that ROS formation accelerates during periods of increased and decreased energy demands (23). Here, we postulated that neurons can manipulate their excitability to minimise the intracellular concentration of ROS. This means that when energetic demands are high, neurons may decrease spiking to avoid FETROS. Conversely, when energetic requirements are too low, metabolic spiking is initiated to produce ADP and relieve RETROS accumulation.

### I. ROS Prevention through Metabolic Spiking.

Unlike other RETROS relieving mechanisms such as ROS scavenging (24), increased lysosomal activity or creatine phosphorylation, (*SI Appendix*, Table S3), metabolic spiking can provide sustained and rapid RETROS relief that may be integrated into the existing set of neuronal heterosynaptic homeostatic mechanisms (42, 43), providing control at fast timescales (44).

The absolute per-spike cost of a neuron is likely to remain constant throughout its lifespan (depending on, e.g., morphology and size of a neuron) and be similar for neurons of the same type and brain region although some modulation may be possible, cf. (45). The non-spiking costs of a neuron, i.e., the position of the star in all figures, can fluctuate widely, for example, due to the synaptic inputs a neuron receives. Hence, the relative metabolic cost of a spike in relation to the ongoing baseline of non-spiking costs in a given neuron will vary and thus affect its effectiveness as a homeostatic measure of intervention (46). What’s more, a neuron’s baseline metabolism may become vulnerable to ROS production in some conditions, or place it permanently in a ROS-safe region (47). Depending on all these factors, different neuron types and even brain regions will be more or less feasible targets to test the above predictions.

We have focused on ROS and ATP as the most pertinent molecules of the neuronal metabolism, but there are likely multiple other contributors that can modulate neuronal spiking, as well as neuronal metabolic activity.

Most notably, we must mention neuronal Ca2+, which can change dramatically due to spiking–regardless of its origin, metabolic or otherwise. In RETROS conditions, Ca2+ influx to mitochondria is rapidly buffered, but the additional divalent cations crossing the mitochondrial membrane would decrease ΔΨ and increase the beneficial effect of metabolic spiking. We have incorporated Ca2+ influx as a direct effect on ΔΨ in the relevant models (Figs. 1 and 2, *Materials and Methods*, and also *SI Appendix*, Fig. S1 *L*–*N*). Ca2+ influx to mitochondria may be mediated by the calcium uniporter (MCU in mode 2) (48–50), which is why we suggested MCU as a possible site of intervention (Fig. 6 *D-5* and *E-5*). On the other hand, the increased synaptic inputs and output spikes in the FETROS regime should lead to greatly increased cytosolic Ca2+ levels. Influx to the mitochondria would be slow but Ca2+ would remain unbound due to saturated mitochondrial buffers (MCU mode 1). Such free Ca2+ could serve as a signal for higher cellular energetic requirements to the mitochondria by enhancing calcium-sensitive mitochondrial matrix enzymes and increase ATP production (48, 49, 51) (and thus temporarily worsen FETROS). Conversely, cytosolic Ca2+ would down-regulate neural excitability through Ca2+ dependent K+ channels and thus decrease spiking and hence limit FETROS. Calcium and other variables (43) could thus be considered part of the metabolic signals that orchestrate the responses to keep neurons safe.

### II. Ion Channels as Metabolic Sensors and Regulators.

The physiological operational regime of neurons between RETROS and FETROS conditions would necessitate that they manipulate their excitability in either direction, presumably by way of several different ion channel types. Metabolic signals may not be limited to ATP, ROS, and Ca2+ but could include signalling molecules in metabotropic cascades (Fig. 3*A* and *SI Appendix*, Table S2). For example, the absence of G${}_{q}$PCR activators could be indicative of lowered input activity and may lead to surplus membrane-bound PIP2, modifying ion channels and thus neuronal excitability (52, 53). Similarly, cyclic-AMP (cAMP) concentration, which is enhanced or diminished by G${}_{s}\alpha$ and G${}_{i}\alpha$ subunits, respectively, can directly affect cAMP-sensitive ion channels such as hyperpolarization-activated cyclic nucleotide–gated channels (HCN) (54, 55).

Metabolic signals may also originate from the pentose phosphate pathway or DNA repair pathways (56) that are favoured under RETROS conditions when excess ATP inhibits glycolysis. Here, a potential candidate metabolic signal is adenosine diphosphate ribose (ADPR) which triggers ribosylation and thus activation of ion channels. Finally, transient extracellular acidification due to more (or fewer) ambient neurotransmitters (glutamate and GABA being abundant and acidic) could be indicative of neural metabolic load and may be detected by extracellular domains of ion channels (such as sodium) changing their pore opening probability.

A special note on the ubiquitous, low-threshold T-type Ca2+ channels may be warranted. Under RETROS conditions, when ROS scavenger pools operate near maximum capacity, the cytosol becomes reduced, thus increasing T-type Ca2+ channel conductivity (57, 58). Increased T-type conductivity in turn facilitates transient UP states that promote bursting and bursting-like firing patterns in accordance with our hypothesis. Importantly, the ion channels that allow for countermeasures against ROS do not have to be de-novo expressions. Instead, they are likely post-translational modifications (e.g., phosphorylation, oxidation, S-glutathionylation, ADP-ribosylation, interactions with pyridine nucleotides, phospholipid docking and so forth) that provide a response to changes in metabolic demands.

### III. Multi-Channel Interactions in an Experimental Study of RETROS.

In the dfb neurons of *D. melanogaster*, ROS levels increase slowly over the duration of the day presumably due to decreasing non-spiking costs nearing sleep (Fig. 4*A*, *B*, and *D* orange to black star). The specific reason for this decrease is as yet unknown and may involve decreased synaptic input to dfb neurons over the day. Adhering to the experimental findings (38), our model only includes the change to the inactivation of the Shaker-Hyperkinetic channels which modulates A-type currents’ domination over non-A-type currents in spike re-polarization. Consequently, the spike frequency adaptation in these neurons is relaxed under RETROS, i.e., the interspike intervals are shortened to accommodate more spikes. Such an interplay between two ion channels, with the only ROS-mediated change involving the inactivation profile of β-subunits, highlights the difficulty of specifying the exact mechanisms involved in metabolic spike generation in every neuron type.

Importantly, the time scale of the metabolic change in these dfb neurons is of the order of several hours. To facilitate a homeostatic response, the specific combination of ion channel and its metabolic signal must operate at speeds compatible with the speed at which non-spiking costs change in the neuron. Depending on a neurons’ placement in a circuit, and its function, we anticipate that a different combination of ion channel changes could achieve similar firing rate changes to serve metabolic homeostasis.

### IV. Sustained and Finely Governed Network Dynamics through Energy Homeostasis.

The activity we simulated is similar to experimentally reported data seen in neural avalanches (12, 40) and in in vivo recordings (59, 60). At the level of populations of neurons, metabolic homeostasis guarantees baseline firing, self-governed network activity near criticality (61), and ensures an essential level of input vigilance. From first impressions, metabolic spiking may seem counterproductive, contributing noise to neural processing. However, such superfluous activity could easily be incorporated into coding schemes, serving to increase the dynamic range and sensitivity of population-based coding by providing non-zero baseline activity (Fig. 5 *C* and *D* black). In previous studies, persistent activity in neural network models could only be guaranteed by way of additional external inputs. The metabolically monitored membrane currents $I_{M}$ make such external inputs unnecessary, as the network becomes self-tuning. Further, spurious spiking of any origin, metabolic or otherwise spontaneous, can be cancelled out in excitation/inhibition balanced networks such that coding is not affected, whereas silent networks would be massively perturbed by initialisation and synchronisation artefacts at the onset of a given stimulus (62). The fine control of persistent activity may promote metabolic spontaneous spiking to also serve crucial integrated functions beyond that of a safety valve preventing ROS toxicity, and it is easy to imagine tasks such as cortical waves (63) or simple background computations rely on consistent baseline activity.

Our hypothesis of metabolic spiking as a means of metabolic homeostasis does not imply that an observed spike is exclusively synaptic or metabolic. Instead, we consider spikes to be agnostic, answering both synaptic and metabolic demands. An auditory neuron spiking at hundreds of Hz (64) without input could be considered in metabolic spiking mode. Upon presentation of relevant input, firing transiently increases, translating synaptic input into rate code. Likewise, in Purkinje cells, neurons exhibit simple and complex spikes (65), which may be considered as metabolic and synaptic respectively. In addition to input integration and metabolic homeostasis, spikes may serve network functions such as a neuromodulatory state. VTA dopaminergic neurons fire at a baseline (metabolic) firing rate that either transiently increases or decreases coding for reward prediction errors (8).

### V. Testable Predictions and Exceptions.

We claimed that when ATP usage is lower than baseline levels, neurons must initiate cellular processes that reduce RETROS or face its toxicity. We proposed a test to verify such a drastic response in Purkinje cells which naturally exhibit high firing rates. Forcing them to resting membrane potential should reveal ROS that is otherwise compensated by metabolic spiking (Fig. 6*A* –*C*), and as such become experimentally observable (66, 67). To establish causality, we also prescribe interventions in the neuron that would decouple mitochondrial metabolism and its excitability (Fig. 6*D*–*F*).

Some of these perturbations have already been performed indirectly in independent studies (*SI Appendix*, Table S1). For example, a connection between the metabolism of a neuron and its spiking output can be gleaned in hippocampal pyramidal neurons of mice, where inhibition of DHODH lowers the firing rate set-point (68). In another study, transient DC hyperpolarizing current which suppressed firing leads to a long-lasting increase in excitability (69).

We expect that exceptions to our hypothesis may apply. For example, we have focused on somatic mitochondria and those in the axon initial segment. In dendrites (70) and axons (71, 72) there may exist other additional mechanisms to cope with ROS locally. More broadly, neural systems that rely on graded potentials, such as retinal photoreceptor cells, as well as neurons in *Caenorhabditis elegans* which also operate with graded potentials for synaptic transmission (73), may not rely on spiking for metabolic homeostasis. Interestingly, *C. elegans* prefer low O2 environments and display an altered sleeping pattern (74), suggesting an entirely different path of ROS homeostasis. More generally, any differences in a neuron’s metabolic profile, its developmental stage, neurotransmitter production, its reliance on astrocytes, as well as its relative location with regard to brain region, blood vessels, and ventricles may also play a role in the unique composition of ion channel expression that dictates a neuron’s response. In that vein, neurons that rarely or never experience RETROS conditions may not have compensatory mechanisms and thus may not initiate metabolic spiking.

In addition to neuronal idiosyncrasies, experimental conditions, such as use of growth factors, temperature-related changes, general slice health, the composition of pipette solutions and artificial cerebrospinal fluid, and even optogenetic stimulation protocols may affect the homeostatic ion concentrations and thus the metabolic status of the neurons. For example, the use of tetrodotoxin (TTX) to block action potential initiation is a common perturbation, but the effects of interactions between the secondary alcohols of TTX and ROS (75, 76) may complicate the interpretation of such experiments in the framework of our hypothesis (See *SI Appendix* for other drugs).

### Further Implications.

Our hypothesis may have some additional implications beyond the supporting models we have presented here. For example, we now know that over their development, neurons undergo a metabolic shift to depend heavily on their mitochondria (34). At such a key transition time point, metabolic spiking may be involved i) in priming cell-specific intrinsic firing patterns, ii) in refining the neural circuits, and iii) in establishing a global firing patterns such as those observed in cortical slow waves.

Metabolic changes in excitability may be directly relevant in behaviour. For example, to set the basal dopamine levels in the striatum. Any decrease in metabolically regulated spiking would therefore result in lowered dopamine levels and may lead to the symptoms in Parkinson’s disease. This is evidenced by the fact that rotenone, an insecticide and herbicide which is known to cause PD-like symptoms, binds to the RETROS release site in the complex 1 of the mitochondria (77), consequently blocking the need to initiate metabolic spiking and thus lowering dopamine levels.

Further, metabolic spiking may also play a role in the onset of some types of epileptic seizures. In our framework, excess run-away activity can be formulated as over-activation of metabolic spiking driven by excess RETROS in neurons or due to hyper-sensitivity of the ion channels sensing the metabolic status. Intriguingly, in some patients where dietary restrictions serve as a therapeutic measure against epilepsy, perhaps the neural metabolism switches in ways that lower homeostatic metabolic spiking and thus seizure vulnerability.

Finally, one may be tempted to speculate that the evolutionary origin of action potentials (a millisecond binary event) may have been driven at least–in part–by metabolic pressures. To quickly process multiple signals from external sources (such as graded potentials or peptidergic inputs) neurons may have evolved toward an on-demand ATP production strategy, utilising a lean ATP production cycle with minimal carbon reserve inventory and an increased dependence on ATP-efficient mitochondria. As such, the baseline energy requirements of the cell could be rapidly met by maintaining a healthy mitochondrion population, and additional ATP can be quickly synthesised when needed. The only caveat of this arrangement is that when ATP usage drops below baseline, cells must be able to deal with imminently changing RETROS conditions. Since any upstream compensation (such as e.g., changing the numbers of mitochondria) would happen at slow timescales, only a transient and immediate increase in ATP consumption could avoid RETROS poisoning. Increasing ATP usage by increasing hyperpolarising leak currents is impractical as it would render the cell unresponsive. Instead, producing a fast and reversible depolarisation to shed ATP, possibly at a specialised (axon initial segment-like) site may have been the preferred strategy. Synaptic integration could well have co-opted this mechanism to transmit outputs as digital signals with better reach, providing the evolutionary advantage of rapidly processing multidimensional spatio-temporal inputs.

### Conclusion.

Metabolic regulation of neural excitability may present a crucial puzzle piece to contextualise the neuronal dynamics that form the basis of all behavior and may have substantial implications for the evolution of the neural code. If true, metabolic spiking adds to the many riches mitochondria bestow upon us.

## Materials and Methods

We modeled the interaction of neural excitability and metabolism in 4 separate models (1 to 4). We started with a mitochondrial metabolism model (37) 1) and included ATP/ROS production dynamics such that

$$\frac{\mathrm{dROS}}{\mathrm{dt}}=\frac{{\mathrm{ROS}}_{\infty }-ROS\left(t\right)}{\tau_{\mathrm{ROS}}},$$

whose steady state $ROS_{\infty }$ is determined by $ATP_{M}$ and ΔΨ values with a cubic relationship.

$${\mathrm{ROS}}_{\infty }\left(ATP_{M},\Delta \Psi \right)=(\frac{ATP_{M}\cdot \Delta \Psi }{f_{\mathrm{RET}}}+\frac{\left(1-ATP_{M}\right)\cdot \left(1-\Delta \Psi \right)}{f_{\mathrm{FET}}}{)}^{3}.$$

Next, we expanded the model to track the cellular metabolic budget and link it to firing properties 2) To demonstrate that even small changes in specific ion channel properties mediated by metabolic (by)products could alter neuronal firing rate, we added metabolic sensing to a single cell 3) and a network model 4) of integrate-and-fire neurons. A more detailed description of the methods can be found in *SI Appendix*.

## Supplementary Material

Appendix 01 (PDF)

## Data Availability

All other data are included in the manuscript and/or *SI Appendix*. No datasets were generated or analysed. Tutorials as well as code for the simulations and for the figures is available at https://github.com/ccluri/metabolic_spiking (78).
