# Supplementary material for: Metabolically regulated spiking could serve neuronal energy homeostasis and protect from reactive oxygen species
Source: Proc Natl Acad Sci U S A. 2023 Nov 21;120(48):e2306525120. doi: 10.1073/pnas.2306525120 (PMC10691349; doi:10.1073/pnas.2306525120)
Supplement: Supplementary file 1 — Appendix 01 (PDF) [file pnas.2306525120.sapp.pdf]

PNAS

Supporting Information for

Metabolically regulated spiking could serve neuronal energy homeostasis and protect from reactive oxygen species

Chaitanya Chintaluri, Tim P. Vogels

Chaitanya Chintaluri.
E-mail: ccluri@gmail.com

This PDF file includes:

Supporting text
Figs. S1 to S6
Tables S1 to S4
SI References

Supporting Information Text

Additional discussion. In this work we build a theoretical framework around the hypothesis of metabolic spiking that is based on two main assumptions listed at the end of the introduction, and inspired by circumstantial experimental evidence; they will (hopefully) serve as hypotheses for future experimental studies, some of which we laid out in Figure 7. We explore the validity of our framework in 4 independent models. In the following, we provide additional considerations and experimental evidence for both assumptions and hypothesis, as well as a detailed Methods section that elaborates on the nature of each model's simplifications.

Mitochondrial reactive oxygen species and their signaling role. ATP production in mitochondria routinely leads to the formation of reactive oxygen species (ROS) (1–3), highly reactive compounds that interfere with cellular processes (4). ROS were initially considered as by-products of metabolism that a cell must cope with, however, in the last two decades, their role in cellular signalling has also gained prominence (5, 6). For instance, ROS has been shown to affect lifespan (7) and a decrease in ROS has been linked with signatures of aging (8). It has also been implicated in heat stress (9, 10), hypoxia (11, 12) and sleep (13, 14). Many other studies also implicate the involvement of ROS in neuro-specific sub-fields, such as in epilepsy (15–17), neurodegeneration (18), ischemia-reperfusion injury (19–21), in neural development (22), and synaptic plasticity (23, 24) to name just a few.

Such a multifaceted role of ROS necessitates more refinement to the specific conditions in which they are produced. As discussed, mitochondrial ROS are produced under two circumstances at the complex I (25). Firstly, "Forward Electron Transport" ROS (FETROS) conditions occur when mitochondria must operate at their maximum capacity (classically known as "respiratory state 3" (26)) and secondly during "Reverse Electron Transport" ROS (RETROS) phases which occur due to mitochondrial ATP production stalling, (classically known as respiratory state 4 (26, 27)). More specifically, FETROS co-occurs with a high flux of glucose through the glycolytic pathway, under oxidised ROS scavenger pools, low cytosolic ATP, and high cytosolic Ca^{2+} concentrations. RETROS co-occurs with low glycolytic flux, reduced scavenger pools, high cytosolic ATP, and low cytosolic Ca^{2+} concentrations.

Fate of glucose in neurons. Glucose is the principal energy source for many cells. The glucose that enters the neurons, crosses the blood-brain barrier and is absorbed via insulin-insensitive glucose transporter 3 (GLUT3) transporters. Cellular glycolysis is regulated by three major enzymes (hexokinase, phosphofructokinase, and pyruvate kinase) (Fig. S5a, b). In neurons, the enzyme hexokinase-1 (HK1) irreversibly phosphorylates glucose into glucose 6-phosphate (G6P), effectively locking glucose inside the cell and committing it to be metabolised (28). Further in neurons, the enzymes phosphofructokinase and pyruvate kinase isozymes M1 are regulated primarily by AMP, ADP, and ATP, with all other regulatory mechanisms nascent (28–33). Such exclusive control allows for a direct link between glycolysis and a neuron's metabolic utility. When ATP demand is high, i.e., in FETROS conditions (Fig. S5a), glucose undergoes glycolysis to produce pyruvate to be further metabolised in the tricarboxylic acid cycle (TCA). On the other hand, when utility of ATP is low, storing glucose as glycogen – like in many other cell types – is generally not observed in healthy neurons (28, 34, 35), and instead the phosphorylated glucose (G6P) may be processed via the pentose phosphate pathway (36) (Fig. S5b).

Pentose phosphate pathway and ROS scavengers. Rerouting glucose via the pentose phosphate pathway (PPP) supports general cellular maintenance through the production of ribose 5-phosphate (R5P) which is used in the synthesis of nucleotides and nucleic acids. These repair pathways are perhaps critical to survival for terminally differentiated post-mitotic cells such as neurons.

More importantly, enhanced PPP has the added benefit of generating reducing equivalents in the form of nicotinamide adenine dinucleotide phosphate (NADPH) (Fig. S5b). NADPH boosts ROS scavenger pools by replenishing the principal redox couples in neurons – glutathione (GSH) and thioredoxin (TXN) (Fig. S5e). Such ROS scavenger pools, through their redox reactions, capture escaped electrons and minimise ROS damage(37). As NADPH's availability is considered to be the limiting step of ROS scavenging in cells(38), any additional pathways that can produce NADPH may also be enhanced if necessary (Fig. S5c).

Under RETROS conditions the scavenger pools operate at their maximum capacity i.e., NADPH produced is fully engaged in scavenging. Consequently, the redox pools persist in their reduced state (Fig. S5e) and any further ROS generated must be quenched by other means due to ROS scavenging overload(37). Conversely, under FETROS conditions, mechanisms for increased ROS scavenging production are less active, as PPP is likely suppressed in favour of energy production via glycolysis. Under these conditions, NADPH supply is limited and consequently, the redox pools are not replenished and remain in their oxidized state (Fig. S5a, d). Here too, ROS generated must be quenched by other means due to low scavenger availability.

Other energy sources in neurons. In addition to the standard metabolism of glucose, neurons utilize amino acids, phosphorylated substrates, glycerol, ketone bodies, etc., for their energy needs (39). Another energy source may be lactate from astrocytes (36, 40, 41). Regardless of the path by which metabolites reach the mitochondria, we argue here that in neurons the metabolic supply chain maintains brim-full proton gradients across the inner mitochondrial membrane, such that the rate-limiting step of ATP production is pushed to the complex V - the last step of ATP production.

For example, cytosolic pyruvate enters the mitochondrial TCA (Fig. S6) where it is converted into acetyl-CoA by unphosphorylated active pyruvate dehydrogenase (PDH). In neurons, PDH remains unphosphorylated (42) due to limited expression of its in-activators – pyruvate dehydrogenase kinase (PDK1, PDK3, and PDK4), and high expression of its

activator pyruvate dehydrogenase phosphatase (PDP1,2)(28, 32). Cytosolic pyruvate can also form malate(43) which enters the mitochondria(44) and can participate in the TCA directly by forming oxaloacetate. In neurons, mitochondrial oxaloacetate either forms alpha-keto glutarate(45, 46) (αKG) or combines with acetyl-CoA to form citrate. Notably, under healthy *in vivo* conditions, due to limited phosphoenolpyruvate carboxykinase(47), (PEPCK) expression oxaloacetate is not turned into PEP. Effectively, this limits the reversal of pyruvate to form glucose and glycogen (34, 35, 48). There is also some evidence suggesting that lactate can directly enter the mitochondria to augment pyruvate (49, 50) (but see(51)).

Additionally, glutamate - the most prevalent neurotransmitter and an abundant amino acid, can also be oxidized to αKG fuelling the TCA and providing metabolic flexibility (52). Similarly, acetyl-CoA could be sourced from other metabolic sources such as ketone bodies from the blood, fatty acids, and leucine. Irrespective of the metabolic sources, nicotinamide adenine dinucleotide (NADH) and flavin adenine dinucleotide ($FADH_2$) produced in the TCA, and other processes such as glycerol 3-phosphate dehydrogenase, electron-transferring flavoprotein (ETF) complex and dihydroorotate dehydrogenase (DHODH) load electrons onto ETC, increasing $\Delta\Psi$, keeping the rate-limiting step of ATP production to complex V and leading to a dearth of ADP, and consequent RETROS as discussed.

Drugs, blockers, and interventions. Pharmacological interventions seem to avail themselves as simple means to test some of the predictions we have put forward here, but some caution may be advised. Ion channel blockers (such as TTX) or receptor blockers for AMPA (CNQX) can cause a variety of changes in metabolic cellular and systems level changes(53).

More generally, any pharmacological perturbations come with some issues. Most importantly, the specificity of a pharmacological blocker is rarely fully resolved, as most drugs bind to several receptors (albeit with varying affinities); as such, many blockers have side effects, e.g., the interaction between TTX and H_2O_2 (a ROS compound) that was mentioned in the main text (54, 55), and CNQX (two other quinoxaline derivatives, NBQX and DNQX) a commonly used AMPA/kainate receptor antagonist were shown to also increase the frequency of spontaneous GABAA receptor-mediated synaptic transmission (53), that would change the metabolic load of a neuron. Similarly, the disruption of membrane-bound lipid rafts by general anesthesia that indirectly increases potassium currents (56) raises some concerns about the secondary and tertiary effects of pharmacological intervention.

For the sake of a thought experiment, let's assume that a blocker like e.g., TTX, through its antagonistic action on a receptor, could decrease the metabolic baseline of a neuron *without* any side effects. How would a neuron respond to this perceived lack of upstream activity? We think it would depend on the neuron and the duration of the perturbation. If decreased baseline consumption, e.g. through input silence on the order of seconds to minutes is routinely encountered by a neuron, we would expect the neuron to spike spontaneously, but not display any other noticeable change. Congruent results have been reported for increased ROS in drosophila melanogaster (13), and we have modelled these results in Fig. 4. However, if such a blockade has never been encountered by a neuron, or is much more profound than what has been experienced in the past (sleep deprivation case, green star in Fig.4d), we would expect that it may push the neuron outside of its operational regime, changing the redox of the cytosol on a timescale of minutes. Consequently, we would expect increases in the following ratios: ATP/ADP, NADH/NAD+, NADPH/NADP+, GSH/GSSH, and CoQH2/CoQ(37). It is more challenging to predict the longer-term consequences on timescales of hours to days. We would not be surprised if some neurons would perish, even if they succeeded in triggering ion channel and receptor synthesis and/or increased trafficking(24) in an attempt to recover their previous homeostatic set-point. Conversely, a permanent absence of ROS in the neuronal metabolism may also lead to functional consequences. In this case, we would expect spontaneous activity to cease altogether.

In the absence of pharmacological blockers without secondary effects, the best location of intervention may thus be intracellular, at the pinch points between metabolism and its downstream signaling cascade (main text, Fig.6). Alternatively, instead of an acute blockage of the receptors, we propose an experiment using CA1 pyramidal neurons, in which AMPA receptors are conditionally knocked out in a small fraction of the cells(57). We speculate that since AMPA receptors are critically necessary to initiate synaptically driven depolarization, NMDA receptors would also be blocked due to Mg+ blockade. According to our hypothesis, we would predict that such neurons, despite lacking AMPA-receptors, and thus means for excitatory synaptic input, will spike spontaneously.

Methods

In this work we began modelling the effect of metabolism on spiking in a mitochondrial model, which we then reproduced - in the abstract - in a metabolic accounting model, showing that its effects could explain experimentally observed firing properties. We demonstrated that even small changes in specific ion channel properties mediated by metabolism could alter neuronal firing rate dramatically. Finally, we added metabolic sensing to a network model of integrate-and-fire neurons, reproducing experimentally observed network dynamics.

Adapted mitochondrial metabolism model. We reproduced and (minimally, see below) adapted a previously published mitochondrial model (58) that simulates the metabolic components of TCA and ATP production in mitochondria with a limited number of differential equations using mass action kinetics and irreversible reactions. The following section follows closely the

appendix A.1 of Nazareth et al., 2009(58)), with a few minor changes. The system evolves as follows:

$$\frac{dp}{d\tau} = v_1 - v_2 - v_7, \qquad \frac{da}{d\tau} = (v_2 - v_3)/\varepsilon_1,$$
$$\frac{dc}{d\tau} = (v_3 - v_4)/\varepsilon_2, \qquad \frac{d\kappa}{d\tau} = (v_4 + v_6 - v_5)/\varepsilon_3,$$
$$\frac{do}{d\tau} = (v_5 + v_7 - v_3 - v_8 - v_6)/\varepsilon_4, \qquad \frac{dn}{d\tau} = (-v_2 - v_4 - 2v_5 + v_{resp})/\varepsilon_5,$$
$$\frac{de}{d\tau} = (v_{ATP} - v_{ANT} + v_5 - v_7)/\varepsilon_6, \qquad \frac{ds}{d\tau} = (10v_{resp} - 3v_{ATP} - v_{leak} - v_{ANT})/\varepsilon_7$$

where the constants $\varepsilon_x, x \in [p, a, c, \kappa, o, n, e, s]$ are ratios of average product concentrations such that

$$\varepsilon_1 = \frac{\overline{AcCoA}}{\overline{Pyr}}, \quad \varepsilon_2 = \frac{\overline{Cit}}{\overline{Pyr}}, \quad \varepsilon_3 = \frac{\overline{\alpha KG}}{\overline{Pyr}}, \quad \varepsilon_4 = \frac{\overline{OAA}}{\overline{Pyr}}, \quad \varepsilon_5 = \frac{N_t}{\overline{Pyr}}, \quad \varepsilon_6 = \frac{A_t}{\overline{Pyr}}, \quad \varepsilon_7 = \frac{\Delta\Psi_m}{\overline{Pyr}}C.$$

p, a, c, κ, o, n, e and s are scaled values of pyruvate (Pyr), acetyl-CoA ($AcCoA$), citrate (Cit), α-ketoglutarate (αKG), oxaloacetate (OAA), NAD+ (NAD), ATP (ATP) and proton gradient ($\Delta\Psi_u$). Their physical values can then be calculated from

$$Pyr = p\overline{Pyr}, \quad AcCoA = a\overline{AcCoA}, \quad Cit = c\overline{Cit}, \quad \alpha KG = \kappa\overline{\alpha KG}, \quad OAA = o\overline{OAA},$$
$$NAD = nN_t, \quad ATP = eA_t, \quad \Delta\Psi_u = s\Delta\Psi_m, \quad t = \tau\frac{\overline{Pyr}}{k_1}$$

with average values as observed in experiments, i.e., $\overline{Pyr} = 0.14$ mM, $\overline{AcCoA} = 0.07$ mV, $\overline{Cit} = 0.4$ mM, $\overline{\alpha KG} = 0.25$ mM and $\overline{OAA} = 0.005$ mM. At these substrate concentrations, $\Delta\Psi_m$, $\overline{ATP}$, $\overline{NAD}$ were measured to be 150 mV, 3.23 mM, and 0.94 mM. Additionally, $N_t = 1.07$ mM and $A_t = 4.16$ mM are the maximum possible concentrations NAD+ and ATP can reach. τ is the scaled time, $C = 6.75$e-6 M/V is the scaled capacitance, and k_1 is the rate constant of pyruvate intake to the model (see below).

Reaction rates $v_x, x \in [1, 2, 3, 4, 5, 6, 7, 8, ANT, ATP, leak, resp]$ were given by

$$v_1 = 1, \quad v_2 = \beta_2 pn, \quad v_3 = \beta_3 oa, \quad v_4 = \beta_4 cn, \quad v_5 = \beta_5 \kappa n(1-e) \quad v_6 = \beta_6(o - \delta_6\kappa),$$
$$v_7 = \beta_7 pe, \quad v_8 = \beta_8 o, \quad v_{ANT} = \beta_{ANT} e, \quad v_{leak} = \beta_{leak} s,$$

$$v_{resp} = \beta_{resp}\frac{1-n}{(\delta_{r1} + 1 - n)}\frac{1}{1 + \exp(\delta_{r2}(s-1))},$$
$$v_{ATP} = \beta_{ATP}\left(\frac{2}{1 + \exp(\delta_{ATP}(e - e_{crit}(s)))} - 1\right),$$
$$\text{where } e_{crit} = \frac{K'_{app}}{K'_{app} + \exp(-\delta_{crit} s)}.$$

The constants β and δ are

$$\beta_2 = \frac{k_2}{k_1}N_t\overline{Pyr}, \quad \beta_3 = \frac{k_3}{k_1}\overline{OAA}\,\overline{AcCoA}, \quad \beta_4 = \frac{k_4}{k_1}N_t\overline{Cit}, \quad \beta_5 = \frac{k_5}{k_1}N_tA_t\overline{\alpha KG}, \quad \beta_6 = \frac{k_6}{k_1}\overline{OAA},$$
$$\beta_7 = \frac{k_7}{k_1}A_t\overline{Pyr}, \quad \beta_8 = \frac{k_8}{k_1}\overline{OAA}, \quad \beta_{ANT} = \frac{k_{ANT}}{k_1}A_t, \quad \beta_{leak} = \frac{k_{leak}}{k_1}\Delta\Psi_m, \quad \beta_{resp} = \frac{k_{resp}}{k_1},$$
$$\beta_{ATP} = \frac{k_{ATP}}{k_1}, \quad \delta_6 = \frac{\overline{\alpha KG}}{\overline{OAA}K_{eq}}, \quad \delta_{r1} = \frac{K_{NAD}}{N_t}, \quad \delta_{r2} = a\Delta\Psi_m, \quad \delta_{ATP} = bA_t, \quad \delta_{crit} = 3\frac{1.2F\Delta\Psi_m}{RT},$$
$$K'_{app} = K_{app}P_i$$

Reaction rates were recorded as $\overline{v_{ATP}} = 174$ μM/s and $\overline{v_{resp}} = 76$ μM/s. Based on these reaction rates, and on the observed equilibrium concentrations of aspartate and glutamate, additional equilibrium constants were set as $K_{app} = 4.6$e-6, $K_{NAD} = 2$ mM, $a = 100$ /V, $b = 4$ /V and $K_{eq} = 0.397$. $P_i = 2.44$ mM is the concentration of phosphate ions, F=96485 C/mol is the Faraday constant, R=8.314 J/(molK) is the gas constant, and T=298 K the temperature in Kelvin, equivalent to 24.8 °C.

In order to obtain a steady state metabolism in the model that is consistent with the experimental literature, i.e., respiratory state 3.5, Nazaret et al. estimated k_1 and k_3 as 38 μM/s and $57,142$ /(Ms), respectively. All other rate constants then follow as $k_2 = 152$ /(Ms), $k_4 = 53$ /(Ms), $k_5 = 82361$ /(M^{2}s), $k_6 = 9.0032$ /s, $k_7 = 40$ /(Ms), $k_8 = 3.6$ /s, $k_{resp} = 2.5$ mM/s, $k_{ATP} = 131.9$ mM/s, $k_{leak} = 0.426$ mM/(Vs) and $k_{ANT} = 0.05387$ /s. In the following, we kept all these rate constants fixed, with the exception of k_{ANT} and k_{leak} where noted.

In the Nazareth model, e, the unitless ATP concentration variable, and n, the unitless NAD concentration variable could assume values $\in$ (0,1). All other variables, p, a, c, κ, o and $s \in [0, \infty)$. Diverging from the Nazaret model, we convert the unitless variable s for the proton gradient to $\Delta\Psi$, such that

$$\Delta\Psi = \frac{(s\Delta\Psi_m) - \Delta\Psi_{min}}{\Delta\Psi_{max} - \Delta\Psi_{min}}. \quad [1]$$

with $\Delta\Psi_{min} = 125$ mV (for k_{ANT}>1 /s) and $\Delta\Psi_{max} = 190$ mV (for k_{ANT}<10^{-3} /s). Assuming that mitochondria operate in the range between these two extreme metabolic states, we can constrain $\Delta\Psi$ to values $\in$ (0, 1). For clarity, we refer to the unitless variable e for mitochondrial ATP concentration as ATP_M ($ATP_M = e$), and we express k_{ANT} in the units of 10^{-3}/s or /ks in the text.

Simplifications in the Nazareth model - (I) The original Nazareth mitochondrial model is a simplified TCA and ATP production model based on mass action kinetics(58). It is constrained by experimentally observed steady-state values of consumption and production in isolated mitochondria. Importantly, the Nazareth model does not include pyruvate production from glycolysis or lactate. **(II)** The Nazareth model also forgoes AMP production or de-novo synthesis. **(III)** Finally, $Ca_2{}^{2+}$ dependent up-regulation of enzymes, creatine phosphorylation, uncoupler proteins, mitochondrial fusion, fission, mitochondrial migration, mitochondrial permeability transition pore opening, and mitophagy are not included in the Nazareth model. These simplifications allow for a focus on the crucial aspects of metabolism relevant to neurons and make the model by Nazareth et al. a good starting point to explore interactions of metabolism and neural excitability.

Integrating mitochondrial metabolism model into a neuron model. To combine mitochondrial ATP production with neural ATP consumption, we considered two variables, one corresponding to the cytosolic ATP level (ATP_C) and the other mitochondrial ATP level (ATP_M). We also considered mitochondria to be the only ATP source[†] such that ATP_M will always be near equilibrium with ATP_C. The non-spiking costs dictate the baseline ATP_C (and hence ATP_M) level. In our model, the non-spiking costs (the position of the $\star$ in all figures) were set by the parameter k_{ANT} that can be assumed to be constant on the time scale of spike-related transients. Low k_{ANT} reflects low exchange of ATP_M with ADP_C, and therefore high levels of ATP_M. Conversely, high k_{ANT} corresponds to low ATP_M and ATP_C levels.

To model calcium influx into the mitochondria after a given spike, we adjust k_{leak}, effectively modulating the leak of protons from the intermembrane space into the matrix $H^+ \xrightarrow{v_{leak}} H_M{}^+$, where $v_{leak} = k_{leak}\Delta\Psi$. Note that Ca^{2+} in the matrix doesn't change any other reaction rates[††]. We consider its efflux to the cytosol to be much slower than the spike-related transients and thus absorbed efflux into the non-spiking costs (k_{ANT}).

Simplifications in the metabolism coupled neuron model - Without loss of generality we can make the following simplifications:**(I)** The mitochondria are the only ATP source and the ATP concentrations in a neuron's cytosol and in its mitochondrial matrix are at equilibrium. **(II)** The Ca^{2+} that enters the mitochondria after each spike is modelled directly as a decrease in $\Delta\Psi$. Ca^{2+} buffering in the mitochondria is not modelled explicitly and its eventual extrusion from the matrix is implicitly included in the non-spiking costs. Due to a lack of neuron-specific data, the regulation of ATP production by free Ca^{2+} in the mitochondria is not included in our model.

Modelling the effect of a spike. The metabolic expense of a spike was modelled as a rapid consumption of ATP_C, followed by a slow return to its baseline, such that $Q_T(f_Q, t)$ is the per-spike cost that occurs at time T (the instance of $V > V_\theta$). We model $Q_T(f_Q, t)$ such that $Q_T(f_Q, t) = f_Q\widetilde{Q_T}(t)$, where $\widetilde{Q_T}(t)$ is an exponentially rising (τ_{Qrise}=5 ms) and falling (τ_{Qfall}=100 ms) function with unit maximum (at t_{lag}), i.e.,

$$\widetilde{Q_T}(t) = \frac{f_T}{max(f_T)} \quad [2]$$

$$f_T(t) = \begin{cases} 0 & \text{t} < T \text{ ms} \\ \frac{1}{\left(\exp\left(\frac{t-1-T}{\tau_{Qfall}}\right)\right) + 30\left(\exp\left(-\frac{t-3-T}{\tau_{Qrise}}\right)\right)} & \text{t} \geq T \text{ ms}, \end{cases} \quad [3]$$

This consumption would appear as a dip in ATP_C such that:

$$ATP_C(t) = ATP_C(k_{ANT}) - Q_T(f_Q, t) \quad [4]$$

where, $ATP_C(k_{ANT})$ is the fixed baseline ATP_C, at k_{ANT} and $Q_T(f_Q, t)$ is the per-spike cost. Consequently, mitochondrial processes will match to produce ATP, i.e., ATP_M will follow ATP_C. In our model, this is achieved by k_{spike} that corrects for the mismatch between ATP_M and ATP_C as follows:

$$k_{spike} = erf(10(ATP_M - ATP_C))/0.1 \quad [5]$$

[†] with near-instantaneous ATP-ADP exchange with the cytosol, a weak assumption under FET, when other sources of ATP like glycolysis or creatine phosphorylation may also be utilised
[††] A weak assumption under FET where $Ca_2{}^+$ buffering and up-regulation of TCA+ETC in neurons is anticipated (59)

Here, erf is the error function. In total, the ATP production in a neuron is $k_{ANT,spike}(t) = k_{ANT} + k_{spike}$, where k_{ANT} is the non-spiking related cost and k_{spike} is the momentary increase of ATP production to reestablish the equilibrium between ATP_C and ATP_M.

The per-spike costs were assumed to include all the metabolic costs incurred after spike initiation, i.e., spike generation, neurotransmitter release, re-uptake, and synaptic vesicle loading. Depending on the modelled neuron type, spike costs vary, which we include as a unitless multiplicative factor f_Q, and expressed as a percentage of maximum ATP, which is normalized to 1 here. Assuming a steady mitochondrion distribution at the spike initiation microdomains, f_Q is fixed[§] for a given neuron. As mentioned above, each spike also initiates $Ca_2{}^+$ entry into the mitochondria (60). As a divalent cation, $Ca_2{}^+$ decreases $\Delta\Psi$, which we model as a transient increase in leak k_{leak}. For simplicity, recovery of k_{leak} follows the same time course as $Q_T(t)$ with a multiplicative factor f_{MCU}, such that $k_{leak,Ca}(t) = k_{leak} + f_{MCU}Q_T(f_Q, t)$.

Unless otherwise mentioned, f_{MCU} is set to 1 mM/Vs. For example, for f_Q= 0.1, post-spike calcium entry to the mitochondria increases k_{leak} from 0.426 mM/s to a maximum of 0.526 mM/s (At t_{lag} from onset) for default f_{MCU}.

Calculating ROS levels. We model ROS levels based on the "redox-optimized ROS balance hypothesis" (37), stating that ROS levels are a function of a cell's redox potential and follow a non-monotonic V-shaped curve (Fig. 1j). The cell's redox can be deduced from the ratios of the so-called redox couples NADH/NAD+, $CoQH_2$/CoQ, GSH/GSSH, NADPH/NADP+, and their respective half-cell reduction potentials. Minimum ROS levels are achieved when all redox couples are balanced, i.e., their ratios approach 1. ROS levels increase at the redox potential extremes, i.e., when the ROS couples approach either the fully reduced or the fully oxidized state.

The oxidised state may be approached due to increased traffic on the ETC during high ATP demands, when the availability of mitochondrial substrates (pyruvate, NADH, and $FADH_2$) becomes rate-limiting. The redox couples are oxidized (i.e. the denominators become bigger than the numerators) and cannot be re-balanced because energy production is prioritised over ROS scavenging, i.e., glucose is routed via glycolysis to pyruvate production instead of the pentose phosphate pathway, hence limiting the scavenging capacity due to low cytosolic NADPH (see below). In other words, ROS production is relatively stable, but scavenging capacity decreases.

The reduced state may be approached when the ETC stalls and ADP in the mitochondria is the rate-limiting step. Here, the redox couples are reduced (i.e. the denominators become smaller than the numerators) and scavengers operate at their maximum capacity. Glucose is routed to the pentose phosphate pathway instead of glycolysis, consequently, cytosolic NADPH production and its utility as a scavenger are at their maximum. In other words, ROS production exceeds the maximum scavenging capacity.

We can express these biochemical relations in mathematical terms as

$$\frac{dROS}{dt} = \frac{ROS_\infty - ROS(t)}{\tau_{ROS}} \qquad [6]$$

whose steady state ROS_∞ is determined by ATP_M and $\Delta\Psi$ values with a cubic relationship.

$$ROS_\infty(ATP_M, \Delta\Psi) = \Big(\frac{ATP_M.\Delta\Psi}{f_{RET}} + \frac{(1 - ATP_M).(1 - \Delta\Psi)}{f_{FET}}\Big)^3, \text{ for } ATP_M \in (0,1), \Delta\Psi \in (0,1) \qquad [7]$$

FETROS and RETROS conditions are thus coupled to the rate at which ATP is produced in the mitochondria as well as to the rate at which ATP is consumed by cellular processes. Under heavy metabolic demands, the ATP consumption is high and any $\Delta\Psi$ is depleted to produce ATP (FETROS). Under low metabolic demands both $\Delta\Psi$ and ATP are high (RETROS). $f_{RET} \in (0, \infty)$ and $f_{FET} \in (0, \infty)$ are tuning parameters that determine the relative amplitudes of RETROS and FETROS production. Unless otherwise mentioned they are set to 1.

Additionally, ROS dynamics are characterised by a time constant (τ_{ROS}) which depends on ATP consumption and peaks near ROS minimum such that

$$\tau_{ROS}(K_{ANT,spike}) = \frac{1200 f_{SCAV}}{\exp\big(4(log(K_{ANT,spike}) - 3.9)\big) + \exp\big(-4(log(K_{ANT,spike}) - 4.6)\big)} \qquad [8]$$

Here, f_{SCAV} is a multiplicative factor that modulates the intensity of scavenging in a neuron. Unless otherwise mentioned we set f_{SCAV} to 1ms. We also tested conditions when τ_{ROS} was kept constant and independent of ATP consumption.

Simplifications in the ROS production model - (I) To express the non-monotonic nature of the ROS levels, we chose a cubic function of normalized ATP and $\Delta\Psi$ values (Eq.7). In real neurons, these steady-state values could be determined by measuring ROS levels while suppressing firing. **(II)** The time constant of the ROS levels serves as a proxy for the ROS scavenger response of the neuron.

Integrating metabolic signal into an accounting model. To explore the hypothesis of ROS homeostasis quantitatively, we first implemented a metabolic accounting model that changes its metabolic signal (MS) according to FETROS and RETROS events such that

[§] but note that f_Q increased when we modelled demyelination or mitochondrial migration away from the spike initiation micro domain

$$\mathrm{MS}(t) = ROS(t) \times \partial ATP(t), \text{where} \quad [9]$$
$$\partial ATP(t) = ATP(t) - \overline{ATP}, \quad [10]$$

with $\overline{ATP} = 0.71$ as the steady state ATP at minimum ROS. We define two thresholds, θ_{RET} and θ_{FET} such that when MS> θ_{RET} the neuron initiates a metabolic spike and enters a refractory period t_{ref}. When MS< θ_{FET}, even synaptically driven spikes cannot be initiated. Two parameters thus dictate the intrinsic firing property landscape: (i) the minimum inter-spike interval between successive spikes t_{ref}, and (ii) the lag time t_{lag} after a spike until the ATP costs affect MS (e.g. related to Na-K pump activation). t_{lag} is defined as the time between spike initiation T and the peak of $Q_T(t)$, and can be varied by changing τ_{Qrise} (eq. 3).

Simplification in accounting model: The metabolic signal (MS) is modelled as the product of ROS levels and ∂ATP. MS is a sufficiently unambiguous signal for the ion channels to gauge whether the neuron is in FETROS or RETROS regime. In reality, multiple ion channels may respond to a metabolic signal (For example, Ca^{2+} or cAMP levels) or to a combination of several metabolic signals (For example, ATP and H_2O_2 levels) that a neuron experiences.

Dorsal fan-shaped body neuron model. As in the experiment, in which it is assumed that the changes in excitation are only due to changes in one ion channel type, we modelled the competitive effects between the A-type and non-A-type K channels on spiking in dfb neurons in a single-compartment model with a single sodium channel type, as well as a delayed rectifier and an A-type potassium channel model. The membrane potential thus follows

$$\frac{\tau_m}{R_m}\frac{dV}{dt} = I_{leak} + I_{Na} + I_K + I_A + I_{clamp},$$

where leak, sodium, delayed rectifier, and A-Type potassium currents can be calculated as

$$\begin{aligned} I_{leak} &= g_{leak}(V_{leak} - V), \\ I_{Na} &= g_{Na}m^3h(E_{Na} - V), \\ I_K &= g_K n^4(E_K - V), \\ I_A &= g_A a^4((1-f)b + f)c(E_K - V), \end{aligned}$$

respectively, and I_{clamp} is the additional externally injected current. Here f is the probability of hyperkinetic β subunits filled with NADP+ (at sleep onset) instead of NADPH (post-sleep). Parameter values V_{leak} =-40 mV, τ_m =100 ms, R_m = 1 GΩ, E_K = -60 mV, E_{Na} =40 mV, g_{leak} =1 nS, g_{Na} =1200 nS, g_K =90 nS, g_A =80 nS are based on typical values (61, 62). The gating variables m, h, n, a, b, and c are standard variables calculated as

$$\frac{dx}{dt} = \frac{x_\infty(V) - x}{\tau_x(V)}$$

where x $\in (m, h, n, a, b, c)$. m,h and n are modified Hodgkin and Huxley variables (61) for Na and K_{DR} channels; the K_A variables a, b and c are based on ion channel studies in ventral cochlear nucleus neurons (62). In detail, the tuned variables present themselves as

$$\begin{aligned} \alpha_m(V) &= 0.1(V+15)/(1-\exp(-0.1(V+15))) & \beta_m(V) &= 4\exp(-0.0556(V+40)) \\ \alpha_h(V) &= 0.07\exp(-0.05(V+40)) & \beta_h(V) &= 1/(1+\exp(-0.1(V+10))) \\ \alpha_n(V) &= 0.003(V+20)/(1-\exp(-0.1(V+20))) & \beta_n(V) &= 0.038\exp(-0.0125(V+30)) \\ m_\infty(V) &= \frac{\alpha_m(V)}{\alpha_m(V)+\beta_m(V)} & \tau_m(V) &= \frac{1}{\alpha_m(V)+\beta_m(V)} \end{aligned}$$

for m,h,and n, and

$$\begin{aligned} a_\infty(V) &= (1/(1+\exp(-(V+6)/6)))^{0.25} & \tau_a(V) &= (100/(7*\exp((V+35)/14)+29*\exp(-(V+35)/24)))+0.1 \\ b_\infty(V) &= (1/(1+\exp((V+41)/7)))^{0.5} & \tau_b(V) &= 0.25(1000/(14\exp((V+35)/27)+29\exp(-(V+35)/24)))+1 \\ c_\infty(V) &= (1/(1+\exp((V+41)/7)))^{0.5} & \tau_c(V) &= 35 \end{aligned}$$

for a,b, and c. Here a is the fast activating gate, b is a fast inactivating gate and c is a slow inactivating gate. Near sleep conditions are modelled as f =0.7. The contribution of the inactivating gate b to the A-type current is less pronounced and thus the channel remains largely non-inactivating. We model awake conditions as f =0 when the b gate rapidly inactivates the A-type current.

Due to a lack of relevant data for the gating variables, ion channel conductances and morphology of this cell, we hand-tuned the gating variables and conductances to match experimental observations(13). We limited manipulations of these ion channels to changes in the inactivation gating variable, but other mechanisms and other ion channels may also be involved in modulating firing in these cells.

Simplification in dFB neuron model - Dorsal fan-shaped body neuron is modelled as a point neuron with three ion channels. Their morphology, channel distribution, and channel properties are not well characterised.

Recurrent network model. To study the effects of metabolic spiking at the network level we constructed a network of 10,000 leaky integrate-and-fire neurons (63). Each integrate-and-fire neuron is characterized by a time constant, $\tau = 20$ ms, and a resting membrane potential, V_{rest}=-60 mV, and a spike threshold, V_θ=-50 mV. After each spike, the membrane potential is clamped at V_{rest} for a refractory period of t_{ref} (see below). To set the scale for currents and conductances in the model, we use a membrane resistance of 100 MΩ. In addition to the standard membrane currents (leak, excitatory, and inhibitory synaptic currents) we added a metabolic current, such that the membrane voltages are calculated as.

$$\tau \frac{dV}{dt} = (V_{rest} - V) + I_{ex} + I_{in} + I_M$$

where

$$I_{ex} = g_{ex}(E_{ex} - V)$$
$$I_{in} = g_{in}(E_{in} - V)$$
$$I_M = \lambda \mathrm{MS}$$

Reversal potentials are $E_{ex} = 0$ mV and $E_{in} = -80$ mV. The synaptic conductances g_{ex} and g_{in} are expressed in units of the resting membrane conductance. Neurons in the network are either excitatory or inhibitory with 4:1 ratio and 2% connectivity. When a neuron fires, the appropriate synaptic variable of its postsynaptic targets is increased, $g_{ex} \to g_{ex} + \Delta g_{ex}$ and $g_{in} \to g_{in} + \Delta g_{in}$ for an excitatory and inhibitory presynaptic neuron, respectively. Otherwise, these parameters obey the following equations:

$$\tau_{ex} \frac{dg_{ex}}{dt} = -g_{ex},$$
$$\tau_{in} \frac{dg_{in}}{dt} = -g_{in},$$

respectively, with $\tau_{ex} = 5$ ms and $\tau_{in} = 10$ ms. I_M, the metabolic current, emulates the combined effect of several ion channels as a current injection of λ that is controlled by the cumulative metabolic signal MS. Here λ, was set to 25 mV. In the absence of an explicit mitochondrial module in our model, MS serves as a proxy for the current ATP consumption rate,

$$\frac{d\mathrm{MS}}{dt} = \frac{\mathrm{MS}_\infty(f) - \mathrm{MS}}{\tau_{\mathrm{MS}}(f)}$$

MS largely depends on the steady-state metabolic consumption MS_∞ due to synaptic inputs f, as well as output spikes (see below), such that

$$\mathrm{MS}_\infty(f) = \left(\frac{2}{1 + \exp\left(-8(f-1)\right)}\right) - 1.$$

The temporal dynamics of MS are governed by a characteristic time constant τ_{MS}

$$\tau_{\mathrm{MS}}(f) = \tau_{Amax} \exp(-(f-1)^2/0.0098) + \tau_{Amin}$$

with $\tau_{Amax} = 1000$ ms and $\tau_{Amin} = 300$ ms which both also depend on f which is expressed as the fraction of synaptic inputs relative to its metabolic optimum L, as follows

$$f(t) = \frac{2L}{L + I_{ex}(t) + |I_{in}(t)|}.$$

The optimal metabolic load number L refers to the point of minimum ROS production in the earlier mitochondrial model above and is used as a neuron-specific, predefined value from a standard normal distribution with mean $\mu = 2$ nA, and standard deviation $\sigma = 0.5$ nA. $|I_{in}|$ is the absolute value of I_{in}. When $I_{ex} + |I_{in}| = L$, $f(t) = 1$, and therefore $\mathrm{MS}_\infty(f) = 0$, and there is no additional metabolic current ($I_M = 0$). When a neuron receives less input than its metabolic optimum L, i.e., $I_{ex} + |I_{in}| < L$, then $f(t) > 1$ and $\mathrm{MS}_\infty(f) > 0$, and therefore $I_M > 0$ – the metabolic current is depolarizing. Conversely, when the neuron receives much larger input than its optimum, i.e., $I_{ex} + |I_{in}| > L$, $f(t) < 1$, $\mathrm{MS}_\infty(f) < 0$, and therefore $I_M < 0$ – the metabolic current becomes hyperpolarizing.

In addition to synaptic inputs, MS is also affected by spikes. To model this effect in integrate and fire neurons, MS is decreased such that $\mathrm{MS} = \mathrm{MS} - q$ after each spike, where q is 0.1 and $t_{ref} = t_{def} - \alpha \mathrm{MS}$, where t_{def} is 5 ms and α is 3 ms and captures changes to spike-adaptation.

Simplification in recurrent neural network model - The metabolic expense of excitatory and inhibitory currents are modelled as equal. In reality, metabolic cost of these currents will depend on the specific composition of the involved ion channels (AMPA vs. NMDA, GABA_A vs. GABA_B).

Software and figures. Simulations were carried out in python(64) using numpy (65), brian (66) and powerlaw(67) packages. Purkinje cell morphology (ID: NMO_10074) was taken from neuromorpho(68). The figures were produced in matplotlib (69) and compiled in libredraw.

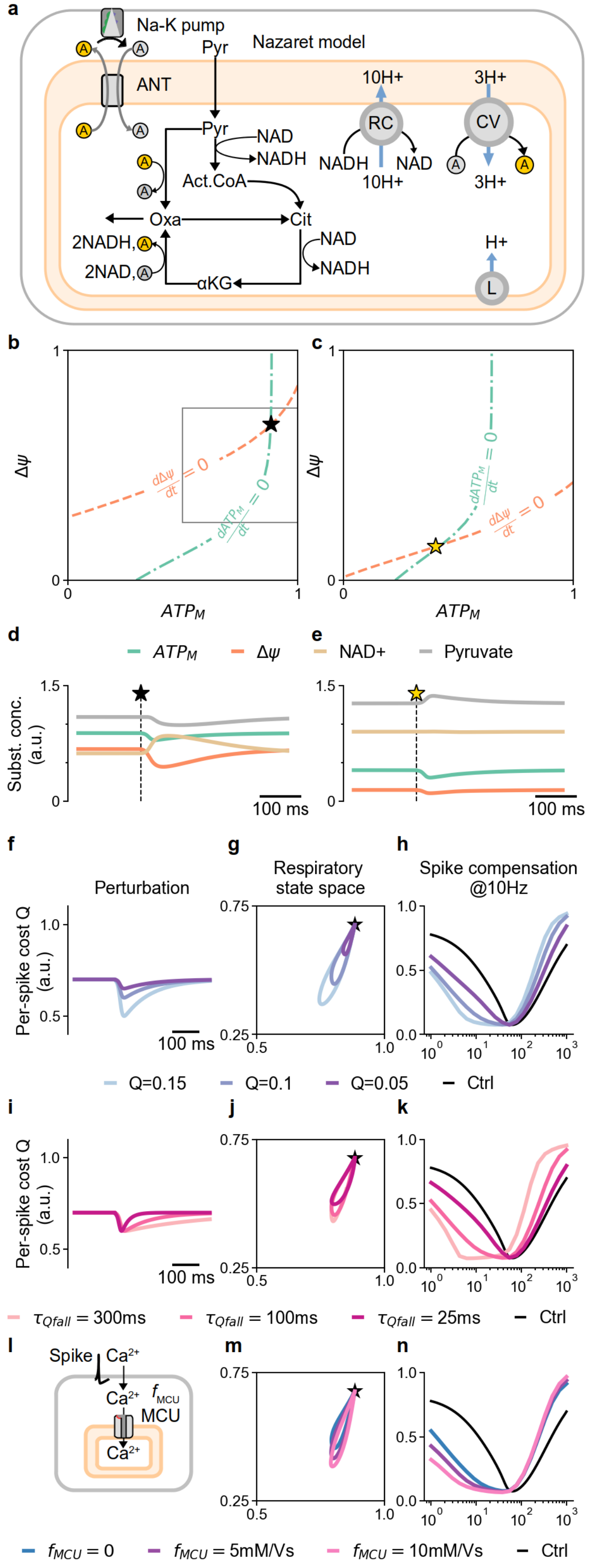

Fig. S1. Physiological factors that affect ROS relief. a) A schematic of a simplified TCA/ETC model for mitochondrial ATP production, i.e., the Nazareth model (58). Here the rate at which ATP$_M$, and ADP$_C$ are exchanged to ADP$_M$ and ATP$_C$ at the adenine nucleotide translocator (ANT), is given by the rate constant k_{ANT} and determines the steady state values of all substrates (in CAPS). **b,c)** k_{ANT} at 30 /ks (black star) and 150 /ks (gold star) corresponds to the fixed points of the nullclines of ATP$_M$ (green) and $\Delta\Psi$ (orange). **d,e)** Changes in the concentrations of all mitochondrial substrates (ATP$_M$ (green), $\Delta\Psi$ (orange), NAD+ - oxidised form of NADH (tan) and Pyruvate (gray, Pyr)) in response to a single spike of cost Q (=0.1).**f)** Examples for perturbations with various values of per-spike cost Q. **g)** Excursions in the respiratory space (inset from b) following perturbations shown in f, starting from the black star. **h)** ROS level compensation due to metabolic spiking at 10 Hz with various Q. **i)** Examples for perturbations with various recovery time constants that change the respiratory steady state excursion **j)** and the amount of ROS relief due to metabolic spiking at 10 Hz **k**. **l)** Schematic of Ca2+ entering the mitochondria following a spike. m) Resulting $\Delta\Psi$ depletion, modelled as an increasing the H+ leak (see a, label L). **m)** ROS relief as a function of Ca2+ entry. **n)** ROS level compensation due to metabolic spiking at 10 Hz for various levels of Ca2+ entry.

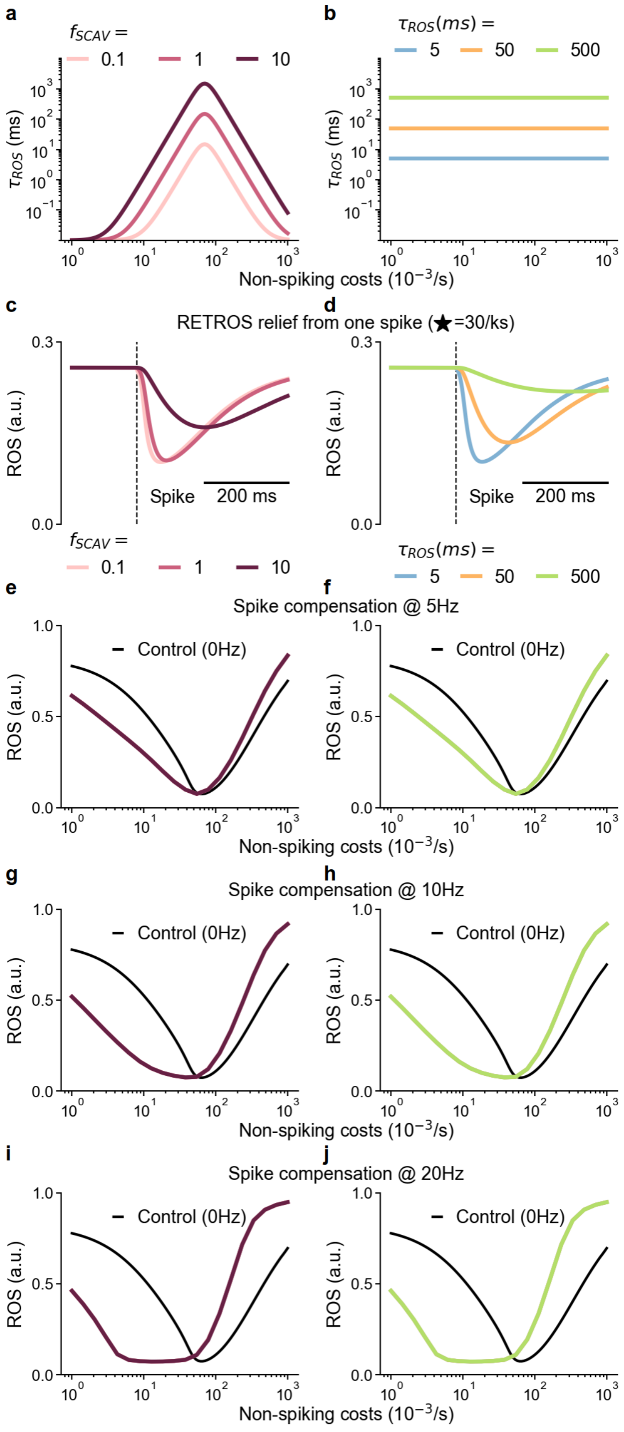

Fig. S2. Time course of ROS relief. The time constant of the ROS accumulation in a neuron depends on its ROS scavenging capacity and may vary depending on the neuron type. Due to a lack of experimental data, we explored a large range of time scales τ_{ROS} for ROS scavenging effectiveness, either $\mathbf{a)}$ as a non-spiking costs dependent function with maximum effectiveness near ROS minimum (pink, red and maroon) or as $\mathbf{b)}$ a constant value (blue, orange and green). The corresponding changes in RETROS relief per spike are shown in $\mathbf{c}$ and $\mathbf{d}$. A single spike occurs at the dotted vertical line. The overall ROS levels change due to spiking at 5 Hz $\mathbf{(e,f)}$, 10 Hz $\mathbf{(g,h)}$ and 20 Hz $\mathbf{(i,j)}$. These results suggest that for a wide range of time scales of ROS accumulation, spiking can affect ROS levels in neurons. Importantly, spikes can provide ROS relief as seen by the collapse of the RETROS levels in plots $\mathbf{e}$-$\mathbf{j}$.

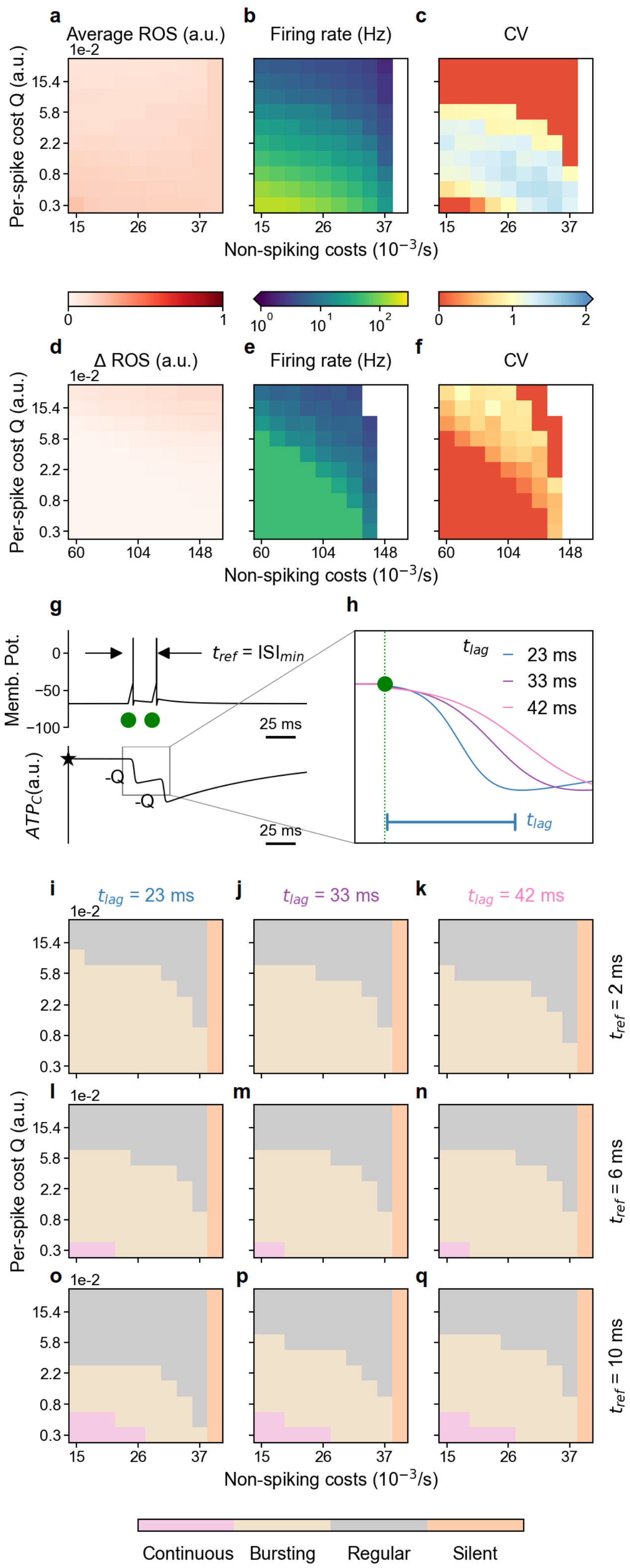

Fig. S3. Summary plots for FETROS and RETROS responses in the accounting model. **a-c)** RETROS response: **a)** Low average ROS levels for metabolic spiking at different levels of baseline ATP consumption and cost-per-spike Q as described in Fig. 2e. **b)** Metabolic firing rate maintaining low ROS levels and **c)** coefficient of variance of the inter-spike intervals (CV ISI) for each parameter pair in a). **d-f)** FETROS response when additional spikes are induced (at 44 Hz and with a CV = 1). **d)** Difference in ROS levels between models with and without a FETROS response. **e)** Resulting firing rates, and **f)** CV ISI for each parameter pair in **d** as described in Fig. 2f. **g)** Schematic of two spikes (top) and the resulting transient decrease in ATP levels (bottom). Green dots denote spike threshold crossing. The minimum interval between crossings is denoted as the refractory period (refrac). **h)** Detail of the fall time of ATP after spike initiation (green dotted line) according to per-spike cost Q. **i-q** Metabolic spike patterns as a function of baseline non-spiking costs (x-axes), per-spike costs Q (y-axes), refractory period (2 ms for **(i,j,k)**, 6 ms for **(l,m,n)**, 10 ms for **(o,p,q)** and time-to-peak of the metabolic response (3 ms for **(i,l,o)**, 5 ms for **(j,m,p)**, 7 ms for **(k,n,q)**).

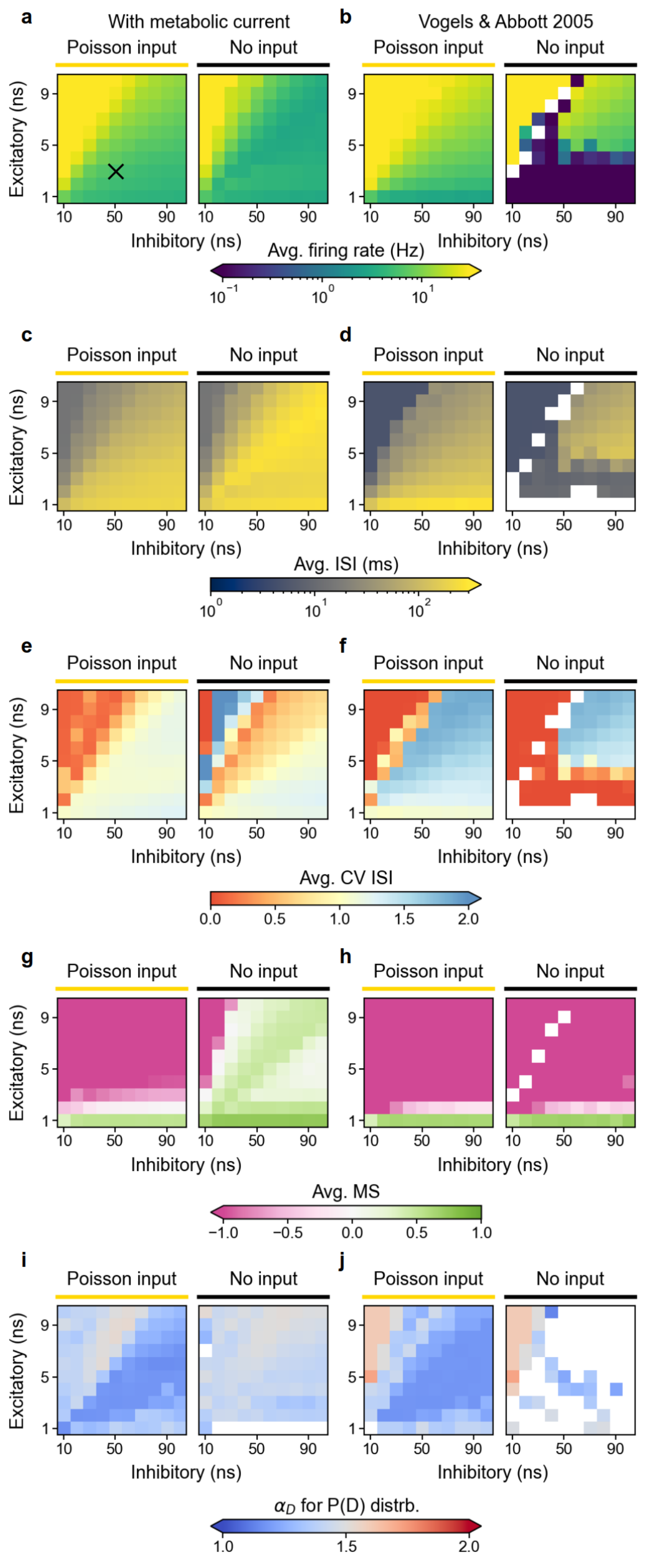

Fig. S4. Summary plots for metabolic recurrent network model. $\mathbf{a,\ c,\ e,\ g,\ i)}$ Recurrent network model with metabolic current compared to the model without $\mathbf{b,\ d,\ f,\ h,\ j)}$. All the values shown here correspond to the averages as indicated by the dotted lines in Fig.3 l,m,n,o, and slope of the line in Fig.3p: $\mathbf{a,\ b)}$ Average firing rates over 5 s simulated time of the network models as a function of inhibitory (x-axes) and excitatory (y-axes) conductance for the cases driven by an external Poisson input of 3 Hz (left), and without any external input (right) The black x in the left-most plot indicates the parameter pair used for simulations in Fig. 5. $\mathbf{c,\ d)}$ Average inter-spike intervals, $\mathbf{e,\ f)}$ average coefficient of variance of the inter-spike intervals (CV ISI) and $\mathbf{g,\ h)}$ the average metabolic signal at the time of spiking. $\mathbf{i,\ j)}$ The slope of the distribution of avalanche durations (1 ms time bins). White patches in these colormaps indicate insufficient data.

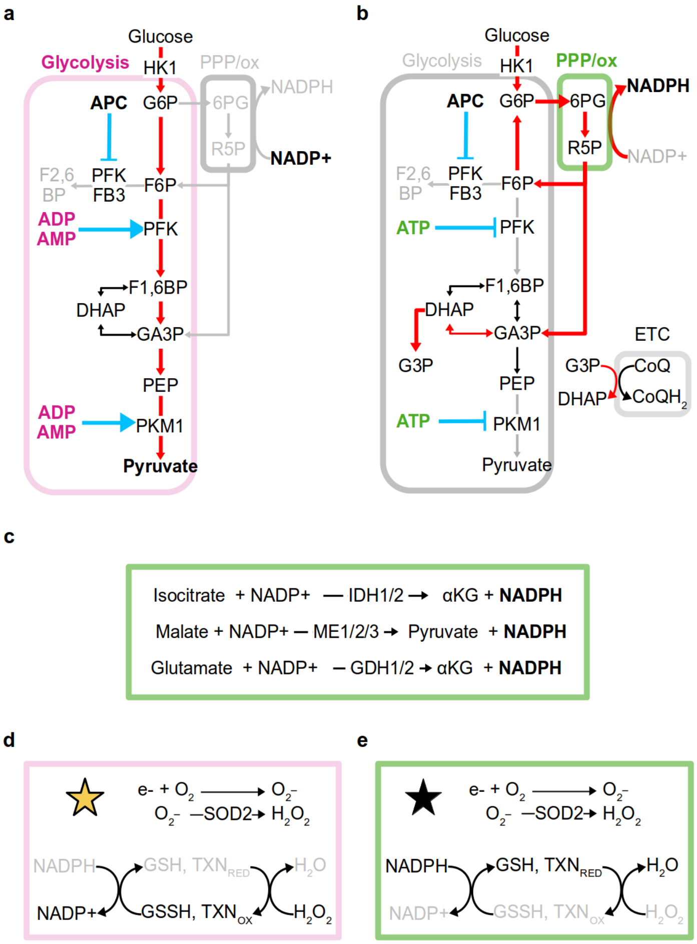

Fig. S5. Overview of neuronal glucose metabolism **a,b)** Neuronal glucose metabolism in RETROS and FETROS conditions, respectively. The preferred pathways for glucose breakdown are shown in red. Neuron-specific regulation is indicated in blue. Unlike in other cell types, the expression of APC inhibits PFKFB3, lowering available F2,6BP. Consequently, neuronal PFK and PKM1 isoforms are primarily regulated by ATP/ADP/AMP. **a)** Under FETROS, ADP/AMP up-regulates glycolysis (pink box) and glucose is diverted away from the PPP/ox (gray box), limiting NADPH production (gray). **b)** Under RETROS conditions, glucose is routed through the oxidative phase of the pentose phosphate pathway (PPP/ox, green box) instead of glycolysis. Consequently, NADPH production is up-regulated (bold), and glycolysis is down-regulated (indicated in gray) by high ATP (bold). **c)** Other reactions that can produce NADPH may also be up-regulated under RETROS. **d, e)** Mitochondrial ROS is scavenged in the cytosol by redox couples GSH/GSSG, thioredoxin (TXN), etc., which are replenished by NADPH. Low and high concentrations are indicated in gray and black respectively. **d)** Under FETROS (gold star), NADPH supply is limited and consequently, the redox pools are mostly oxidized. **e)** Under RETROS conditions (black star) the scavenger pools operate at their maximum capacity and are mostly reduced.

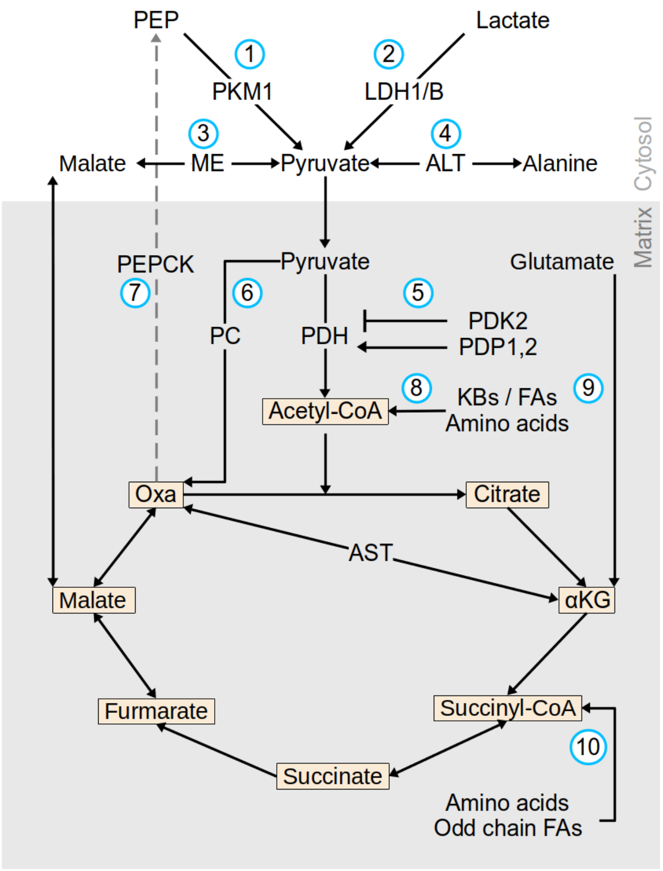

Fig. S6. Overview of neuronal metabolic flexibility Metabolic pathways in neurons may operate flexibly to percolate metabolic fuels perpetually into the mitochondria, thus maintaining a high $\Delta\Psi$. Neuron-specific pathways are indicated in blue circled numbers and citric acid cycle (TCA) substrates are shown in peach-colored boxes. Cytosolic pyruvate in neurons can be produced from glycolysis, where phosphoenolpyruvate (PEP) is converted to pyruvate. In neurons, this step is regulated by PKM1 isoform (1) which lacks allosteric regulation by F1,6BP and alanine. Pyruvate may also be produced from lactate (2), from malate (3), and from some amino acids such as alanine (4). Mitochondrial pyruvate enters the TCA as acetyl-CoA by the pyruvate dehydrogenase (PDH). In neurons, PDH perhaps largely remains in its active state due to low expression of its inhibitory regulator (5) pyruvate dehydrogenase kinases (PDK1,3,4) and high expression of its activator pyruvate dehydrogenase phosphatase (PDP1,2). Pyruvate may also enter the TCA indirectly as (6) oxaloacetate (Oxa). Oxa (and glutamate) can be converted to aspartate (and αKG) instead of PEP due to limited phosphoenolpyruvate carboxykinase (PEPCK) expression (7, gray dashed line). Acetyl-CoA itself can also be augmented by (8) ketone bodies (KBs), fatty acids (FAs) and amino acids. Additionally, glutamate (9) - the most abundant free amino acid in the brain, can also enter TCA via αKG. Finally, odd chain FAs and some amino acids (10) can also produce TCA intermediate succinyl-CoA.

Table S1. Experimental observations in agreement with the intrinsic excitability hypothesis.

No.	*Perturbation*	*Effect*	*Reference*
1	miniSOG/AOX	Genetically-encodable light-induced stimulation of mini singlet oxygen generator (miniSOG) adds cytosolic ROS and increases firing rate. Conversely, expressing plant mitochondrial alternative oxidase (AOX) lowers $CoQH_2$ pools in ETC and decreases the firing rate of dorsal fan-shaped body neurons in fruit flies, changing their sleep state	(13).
2	DHODH	Mitochondrial protein dihydroorotate dehydrogenase (DHODH) involved in de novo pyrimidine biosynthesis also increases $CoQH_2$ pools in the ETC. Inhibition lowers the firing rate set-point in CA1 pyramidal neurons.	(17)
3	Low lactate	Lactate dehydrogenase in neurons produces pyruvate from lactate. Its inhibition is reported to lower excitability in neurons and suppress seizures in an in vivo mouse model for epilepsy.	(70)
4	Pipette	Increased excitability of neurons in slice compared to in vivo seen in layer 2/3 pyramidal neurons. This is potentially due to a decrease in the long-range network connectivity and therefore decrease in non-spiking costs.	(71)
5	DC hyperpolarization	Prolonged DC current injection to inhibit spontaneous firing induces long-lasting increase in excitability. Prohibiting neurons from firing can increase ETC stalling (more $CoQH_2$ pools) and may enhance the homeostatic metabolic spiking.	(72)
6	Creatine deficiency	Mutations in the SLC6A8 gene can cause X-linked creatine transporter deficiency with its hallmark symptoms including eplilepsy. Lack of creatine in neurons would lead to lowered ATP buffering and may prompt increases in metabolic spiking and thus vulnerability to seizure onsets.	(73)
7	Low-carb diet	Diets low in carbohydrates have long been prescribed as countermeasures to some drug-resistant epilepsy. Here, neurons may rely more on differently regulated ketone bodies and amino acids for their metabolic needs and decrease their dependence on pyruvate. Perhaps this changes the neuronal metabolism in ways that lead to fewer metabolic spikes and thus lower seizure onset	(74, 75)

Table S2. Potential ion channels and their corresponding metabolic signals that align with the intrinsic excitability hypothesis.

Ion channel	*Metabolic signal*	*Notes*	*Reference*
**ATP spenders**			
Depolarizers			
Na_P (persistent)	Hypoxia, GSH	Hypoxia and GSH increase the conductivity of TTX and lidocaine sensitive non-inactivating persistent sodium current. NaCN, DTT also induces this effect. Observed in hippocampal neurons.	(76, 77)
	H_2O_2	Competitive changes between Na_T and Na_P due to H_2O_2. Na_T inactivation shifts to more negative membrane potentials.	(78, 79)
	unknown	$\beta 2$, $\beta 3$ and $\beta 4$ facilitate persistent sodium currents. $\beta 1$ can modulate $\beta 4$	(80, 81)
Na_T (transient)	extracellular pH	Alkaline conditions hyperpolarize the activation profile of NaV1.1-3,5. Inactivation is also hyperpolarized for NaV1.5. These conditions may occur due to diminished acidic neurotransmitters in the extracellular space.	(82, 83)
	PKA, PKC	Phosphorylation of α subunits lowers the peak Nav1.2 channel current. The opposite condition may arise due to a lack of G_sPCR or G_qPCR activators.	(84)
	Met residues	Oxidation of methionine residues in the linker between D3-D4 removes inactivation.	(85)
TRPM2	ADPR + H_2O_2	ADPR originating from mitochondria binds to the NUDT9H domain to activate. H_2O_2 increases conductivity.	(86–89)
TRPC3/5	PIP_2, DAG	PIP_2 and DAG required for activation. Low G_qPCR activators favour these conditions. Here, the role of DAG is unclear.	
HCN (pacemaker)	cAMP	Support high firing by shifting activation towards depolarizing potentials due to increased cAMP which may occur due to lack of G_iPCR activators and excess ATP.	(90, 91)
Adaptation modifiers			
KA	Cys residues	Modification of cystine (disulfide bridges) residues on N-termial slows N-type inactivation.	(92)
	aldo-keto reductase	β-subunits of Kv1 also function as aldo-keto reductase and catalyze NADPH bound to β-subunit to NADP+ and weaken inactivation.	(93)
	PIP_2	PIP_2 prevents N-type inactivation Kv1.1+Kvβ1.1 channels. This condition may occur due to a lack of G_qPCR activators.	(94)
BK (Maxi)	redox	Reducing agents shift activation towards depolarizing potentials. These conditions are favoured under RETROS.	(95? –97)
	PIP_2	PIP_2 enhances Ca^{2+}-driven gating channel conductivity. This condition may occur due to a lack of G_qPCR activators.	(98, 99)
Up-state inducers			
Ca_T	redox	Reducing agents enhance CaV3.2 and oxidizing agents inhibit Cav3.1,2,3. Reduced cytosolic conditions can occur under RETROS conditions.	(100, 101)
	PKA	cAMP-dependent PKA enhance Cav3.2 currents. This condition may arise during lack of G_iPCR activators.	(102)
Refractory removers			
Na_R (resurgent)	unknown	$\beta 4$-peptide acts as an open channel blocker and prevents fast inactivation. Phosphotases abolishes this blocking function.	(103–105)
Other			
RyR	PIP_2	Ryanodine receptors channels facilitate the release of Ca_2^+ from the sarcoplasmic reticulum.	(106)
NCX	PIP_2	PIP_2 binds to XIP region of sodium/calcium exchanger (NCX) and removes inactivation. $3Na^+$ exchanged for $1Ca^{2+}$.	(107)
**ATP savers**			
K_{ATP}:SUR1	ATP, H_2O_2	Activates with low ATP or high H_2O_2 to prevents spiking.	(108)
K_{ATP}:SUR2	ATP	Prevents spiking under low ATP.	(108)
SK	Ca^{2+}	Modulates excitability due to an increase in intracellular Ca_2^+ that binds to calmodulin in the c-terminal.	(109)
TRPM2	ADPR + AMP	Decreases conductivity with increasing AMP. A condition that may occur in FETROS.	(110)
TRPC3/5	PKC	G_qPCR activation favours increases in PKC which in turn inhibits these channels.	(111)

Table S3. In addition to modulating intrinsic excitability in neurons, other potential ROS-relieving mechanisms that maybe enhanced during RETROS or FETROS conditions.

	Process	*Notes*	*Reference*
**RETROS**			
Pre complex V			
	Pentose phosphate pathway	Up-regulation and consequent ribose 5-phosphate production may promote repair pathways and protein synthesis.	(112, 113)
	Glycogen synthesis	Glucose is stored as glycogen in astrocytes instead of glycolysis.	(114, 115)
	Uncoupler proteins	Action of UCP2, UCP4 and UCP5 in the mitochondria, can decreases $\Delta\Psi$. However, they also import fatty acids and amino acids and despite their action the pool of $CoQH_2$ on ETC remains unchanged.	(116–118)
Post complex V			
	Creatine phosphorylation	Phosphorylation of creatine uses ATP to produce phosphocreatine.	(119)
	Lysosomal activity	Favourable conditions for vacuolar-type ATPase-dependent lysosomal acidification.	(120)
	Synaptic vesicle	Favourable conditions for acidification of synaptic vesicle pools.	(121)
	Synaptic changes	Spine changes, vesicle formation, and neurotransmitter synthesis.	(122)
	Ca^{2+} clearance	Ca^{2+} buffered in organelle may be cleared from the cell.	(107)
	Mitochondrial bio-genesis	Favourable conditions for mitochondrial fission and fusion.	(123, 124)
	Cellular cargo transport	Promotion of cytoskeletal motor proteins (dynein and kinesin).	(125)
**FETROS**			
Pre complex V			
	Glycolysis up regulation	PFK1 and PKM1 are allosterically activated by AMP and may up-regulate glycolysis to produce more ATP.	(126, 127)
	Glycogen metabolism	To meet the energy demands of neurons, the metabolism of glycogen to glucose in astrocytes may be enhanced.	(128, 129)
Post complex V			
	Creatine de-phosphorylation	Free ATP is produced when creatine is de-phosphorylated. This is limited by the availability of phosphorylated creatine.	(119)
	Lipid transfer	Excess fatty acids generated under high energy demands would be transported to astrocytes to limit further ROS.	(130)

Table S4. Comparison of metabolism between neurons, astrocytes and muscle cells. All these cells are terminally differentiated.

Process	*Neurons*	*Astrocytes*	*Type 1 muscle*	*Type 2A muscle*
Nutrient source	BBB, astrocytes	BBB, capillary	capillary	capillary
Aerobic	yes	yes	yes	yes
Anaerobic	no	yes	no	yes
Oxidative phosphorylation	yes	low	yes	yes
Primary fuels	lactate, glucose	glucose	glucose	glucose
Fatty acid fuels	yes	long-chain	yes	yes
Amino acid fuels	yes	yes	yes	yes
Ketone bodies	yes	yes	yes	yes
Creatine	yes	yes	low	yes
Carbohydrate reserves	none	glycogen, lipids	triglycerides	glycogen
O_2 homeostasis	neuroglobin	neuroglobin	myoglobin (high)	myoglobin (low)
Primary glucose intake	GLUT3	GLUT1	GLUT4	GLUT4
Extracellular glucose conc.	2-3mM	2-3mM	6-7mM	6-7mM
Insulin-glucose uptake	largely insensitive	largely insensitive	yes	yes
Metabolic end products	ATP	lactate, ATP, glutamine	ATP	ATP, lactate
Primary ATP consumer	ion pumps	ion pumps, glutamine synthetase	myosin ATPase	myosin ATPase
Multi-nucleated	no	no	yes	yes

References

1. PK Jensen, Antimycin-insensitive oxidation of succinate and reduced nicotinamide-adenine dinucleotide in electron-transport particles I. pH dependency and hydrogen peroxide formation. *Biochimica et Biophys. Acta (BBA) - Enzymol. Biol. Oxid.* **122**, 157–166 (1966).
2. G Loschen, L Flohé, B Chance, Respiratory chain linked H2O2 production in pigeon heart mitochondria. *FEBS Lett.* **18**, 261–264 (1971).
3. A Boveris, B Chance, The mitochondrial generation of hydrogen peroxide. General properties and effect of hyperbaric oxygen. *Biochem. J.* **134**, 707–716 (1973).
4. RL Auten, JM Davis, Oxygen Toxicity and Reactive Oxygen Species: The Devil Is in the Details. *Pediatr. Res.* **66**, 121–127 (2009) Number: 2 Publisher: Nature Publishing Group.
5. CR Reczek, NS Chandel, ROS-dependent signal transduction. *Curr. Opin. Cell Biol.* **33**, 8–13 (2015).
6. H Sies, DP Jones, Reactive oxygen species (ROS) as pleiotropic physiological signalling agents. *Nat. Rev. Mol. Cell Biol.* **21**, 363–383 (2020) Bandiera_abtest: a Cg_type: Nature Research Journals Number: 7 Primary_atype: Reviews Publisher: Nature Publishing Group Subject_term: Cell signalling;Mechanisms of disease Subject_term_id: cell-signalling;mechanisms-of-disease.
7. F Scialò, et al., Mitochondrial ROS Produced via Reverse Electron Transport Extend Animal Lifespan. *Cell Metab.* **23**, 725–734 (2016).
8. C Graham, et al., ROS signalling requires uninterrupted electron flow and is lost during ageing in flies, Technical report (2021).
9. F Scialò, et al., Mitochondrial complex I derived ROS regulate stress adaptation in Drosophila melanogaster. *Redox Biol.* **32**, 101450 (2020).
10. SA Dogan, et al., Perturbed Redox Signaling Exacerbates a Mitochondrial Myopathy. *Cell Metab.* **28**, 764–775.e5 (2018) Publisher: Elsevier.
11. MC Fernández-Agüera, et al., Oxygen Sensing by Arterial Chemoreceptors Depends on Mitochondrial Complex I Signaling. *Cell Metab.* **22**, 825–837 (2015) Publisher: Elsevier.
12. F Bergmann, BU Keller, Impact of mitochondrial inhibition on excitability and cytosolic Ca2+ levels in brainstem motoneurones from mouse. *J. Physiol.* **555**, 45–59 (2004).
13. A Kempf, SM Song, CB Talbot, G Miesenböck, A potassium channel -subunit couples mitochondrial electron transport to sleep. *Nature* **568**, 230–234 (2019).
14. S Dissel, MK Klose, Bv Swinderen, L Cao, PJ Shaw, Sleep promoting neurons remodel their response properties to calibrate sleep drive with environmental demands, Technical report (2021).
15. Y Zhang, et al., Succinate accumulation induces mitochondrial reactive oxygen species generation and promotes status epilepticus in the kainic acid rat model. *Redox Biol.* **28**, 101365 (2019).
16. S Waldbaum, M Patel, Mitochondria, oxidative stress, and temporal lobe epilepsy. *Epilepsy Res.* **88**, 23–45 (2010).
17. B Styr, et al., Mitochondrial Regulation of the Hippocampal Firing Rate Set Point and Seizure Susceptibility. *Neuron* **102**, 1009–1024.e8 (2019) Publisher: Elsevier Inc.
18. YM Lee, W He, YC Liou, The redox language in neurodegenerative diseases: oxidative post-translational modifications by hydrogen peroxide. *Cell Death & Dis.* **12**, 1–13 (2021) Bandiera_abtest: a Cc_license_type: cc_by Cg_type: Nature Research Journals Number: 1 Primary_atype: Reviews Publisher: Nature Publishing Group Subject_term: Neurodegenerative diseases;Post-translational modifications Subject_term_id: neurodegenerative-diseases;post-translational-modifications.
19. T Ross, et al., Reverse electron flow-mediated ROS generation in ischemia-damaged mitochondria: role of complex I inhibition vs. depolarization of inner mitochondrial membrane. *Biochimica Et Biophys. Acta* **1830**, 4537–4542 (2013).
20. ET Chouchani, et al., Ischaemic accumulation of succinate controls reperfusion injury through mitochondrial ROS. *Nature* **515**, 431–435 (2014) Bandiera_abtest: a Cg_type: Nature Research Journals Number: 7527 Primary_atype: Research Publisher: Nature Publishing Group Subject_term: Metabolomics Subject_term_id: metabolomics.
21. AS Milliken, CA Kulkarni, PS Brookes, Acid enhancement of ROS generation by complex-I reverse electron transport is balanced by acid inhibition of complex-II: Relevance for tissue reperfusion injury. *Redox Biol.* **37**, 101733 (2020).
22. MCW Oswald, N Garnham, ST Sweeney, M Landgraf, Regulation of neuronal development and function by ROS. *FEBS Lett.* **592**, 679–691 (2018) _eprint: https://onlinelibrary.wiley.com/doi/pdf/10.1002/1873-3468.12972.
23. MC Oswald, et al., Reactive oxygen species regulate activity- dependent neuronal plasticity in Drosophila. *eLife* **7** (2018).
24. RL Doser, GC Amberg, FJ Hoerndli, Reactive Oxygen Species Modulate Activity-Dependent AMPA Receptor Transport in C. elegans. *J. Neurosci.* **40**, 7405–7420 (2020) Publisher: Society for Neuroscience Section: Research Articles.
25. MP Murphy, How mitochondria produce reactive oxygen species. *Biochem. J.* **417**, 1–13 (2009).
26. B Chance, GR Williams, Respiratory enzymes in oxidative phosphorylation. III. The steady state. *The J. Biol. Chem.* **217**, 409–427 (1955).
27. B Chance, G Hollunger, The interaction of energy and electron transfer reactions in mitochondria. I. General properties and nature of the products of succinate-linked reduction of pyridine nucleotide. *The J. Biol. Chem.* **236**, 1534–1543 (1961).
28. X Zheng, et al., Metabolic reprogramming during neuronal differentiation from aerobic glycolysis to neuronal oxidative phosphorylation. *eLife* **5**, e13374 (2016) Publisher: eLife Sciences Publications, Ltd.

29. JD Dombrauckas, BD Santarsiero, AD Mesecar, Structural basis for tumor pyruvate kinase M2 allosteric regulation and catalysis. *Biochemistry* **44**, 9417–9429 (2005).
30. JP Bolaños, A Almeida, The pentose-phosphate pathway in neuronal survival against nitrosative stress. *IUBMB Life* **62**, 14–18 (2010).
31. HP Morgan, et al., M2 pyruvate kinase provides a mechanism for nutrient sensing and regulation of cell proliferation. *Proc. Natl. Acad. Sci.* **110**, 5881–5886 (2013) Publisher: National Academy of Sciences Section: Biological Sciences.
32. Y Zhang, et al., An RNA-Sequencing Transcriptome and Splicing Database of Glia, Neurons, and Vascular Cells of the Cerebral Cortex. *The J. Neurosci.* **34**, 11929–11947 (2014).
33. M Yuan, et al., An allostatic mechanism for M2 pyruvate kinase as an amino-acid sensor. *Biochem. J.* **475**, 1821–1837 (2018).
34. AM Cataldo, RD Broadwell, Cytochemical identification of cerebral glycogen and glucose-6-phosphatase activity under normal and experimental conditions. II. Choroid plexus and ependymal epithelia, endothelia and pericytes. *J. Neurocytol.* **15**, 511–524 (1986).
35. D Vilchez, et al., Mechanism suppressing glycogen synthesis in neurons and its demise in progressive myoclonus epilepsy. *Nat. Neurosci.* **10**, 1407–1413 (2007).
36. PJ Magistretti, I Allaman, A Cellular Perspective on Brain Energy Metabolism and Functional Imaging. *Neuron* **86**, 883–901 (2015) Publisher: Elsevier Inc.
37. MA Aon, S Cortassa, B O'Rourke, Redox-optimized ROS balance: A unifying hypothesis. *Biochimica et Biophys. Acta - Bioenerg.* **1797**, 865–877 (2010) Publisher: Elsevier B.V.
38. W Xiao, RS Wang, DE Handy, J Loscalzo, NAD(H) and NADP(H) Redox Couples and Cellular Energy Metabolism. *Antioxidants Redox Signal.* **28**, 251–272 (2018) Publisher: Mary Ann Liebert Inc.
39. S Camandola, MP Mattson, Brain metabolism in health, aging, and neurodegeneration. *The EMBO J.* **36**, 1474–1492 (2017) Publisher: John Wiley & Sons, Ltd.
40. PG Bittar, Y Charnay, L Pellerin, C Bouras, PJ Magistretti, Selective distribution of lactate dehydrogenase isoenzymes in neurons and astrocytes of human brain. *J. Cereb. Blood Flow Metab.* **16**, 1079–1089 (1996).
41. PJ Magistretti, I Allaman, Lactate in the brain: from metabolic end-product to signalling molecule. *Nat. Rev. Neurosci.* **19**, 235–249 (2018) Publisher: Nature Publishing Group.
42. ND HALIM, et al., Phosphorylation Status of Pyruvate Dehydrogenase Distinguishes Metabolic Phenotypes of Cultured Rat Brain Astrocytes and Neurons. *Glia* **58**, 1168–1176 (2010).
43. B Hassel, Pyruvate carboxylation in neurons. *J. Neurosci. Res.* **66**, 755–762 (2001).
44. MC McKenna, HS Waagepetersen, A Schousboe, U Sonnewald, Neuronal and astrocytic shuttle mechanisms for cytosolic-mitochondrial transfer of reducing equivalents: Current evidence and pharmacological tools. *Biochem. Pharmacol.* **71**, 399–407 (2006).
45. G Palaiologos, L Hertz, A Schousboe, Evidence that aspartate aminotransferase activity and ketodicarboxylate carrier function are essential for biosynthesis of transmitter glutamate. *J. Neurochem.* **51**, 317–320 (1988).
46. MC McKenna, IB Hopkins, SL Lindauer, P Bamford, Aspartate aminotransferase in synaptic and nonsynaptic mitochondria: differential effect of compounds that influence transient hetero-enzyme complex (metabolon) formation. *Neurochem. Int.* **48**, 629–636 (2006).
47. F Cruz, SR Scott, I Barroso, P Santisteban, S Cerdán, Ontogeny and Cellular Localization of the Pyruvate Recycling System in Rat Brain. *J. Neurochem.* **70**, 2613–2619 (1998) _eprint: https://onlinelibrary.wiley.com/doi/pdf/10.1046/j.1471-4159.1998.70062613.x.
48. M Cesar, B Hamprecht, Immunocytochemical examination of neural rat and mouse primary cultures using monoclonal antibodies raised against pyruvate carboxylase. *J. Neurochem.* **64**, 2312–2318 (1995).
49. T Hashimoto, R Hussien, HS Cho, D Kaufer, GA Brooks, Evidence for the Mitochondrial Lactate Oxidation Complex in Rat Neurons: Demonstration of an Essential Component of Brain Lactate Shuttles. *PLOS ONE* **3**, e2915 (2008) Publisher: Public Library of Science.
50. A Young, C Oldford, RJ Mailloux, Lactate dehydrogenase supports lactate oxidation in mitochondria isolated from different mouse tissues. *Redox Biol.* **28**, 101339 (2020).
51. B Glancy, et al., Mitochondrial lactate metabolism: history and implications for exercise and disease. *The J. Physiol.* **599**, 863–888 (2021) _eprint: https://onlinelibrary.wiley.com/doi/pdf/10.1113/JP278930.
52. AS Divakaruni, et al., Inhibition of the mitochondrial pyruvate carrier protects from excitotoxic neuronal death. *J. Cell Biol.* **216**, 1091–1105 (2017).
53. SG Brickley, M Farrant, GT Swanson, SG Cull-Candy, CNQX increases GABA-mediated synaptic transmission in the cerebellum by an AMPA/kainate receptor-independent mechanism. *Neuropharmacology* **41**, 730–736 (2001).
54. R Chicheportiche, M Balerna, A Lombet, G Romey, M Lazdunski, Synthesis of new, highly radioactive tetrodotoxin derivatives and their binding properties to the sodium channel. *Eur. J. Biochem.* **104**, 617–625 (1980).
55. AK Chauhan, NS Magoski, Hydrogen Peroxide Gates a Voltage-Dependent Cation Current in Aplysia Neuroendocrine Cells. *J. Neurosci.* **39**, 9900–9913 (2019) Publisher: Society for Neuroscience Section: Research Articles.
56. MA Pavel, EN Petersen, H Wang, RA Lerner, SB Hansen, Studies on the mechanism of general anesthesia. *Proc. Natl. Acad. Sci.* **117**, 13757–13766 (2020).
57. W Lu, et al., Subunit Composition of Synaptic AMPA Receptors Revealed by a Single-Cell Genetic Approach. *Neuron*

62, 254–268 (2009).

58. C Nazaret, M Heiske, K Thurley, JP Mazat, Mitochondrial energetic metabolism: A simplified model of TCA cycle with ATP production. *J. Theor. Biol.* **258**, 455–464 (2009).
59. G Ashrafi, Jd Juan-Sanz, RJ Farrell, TA Ryan, Molecular Tuning of the Axonal Mitochondrial Ca2+ Uniporter Ensures Metabolic Flexibility of Neurotransmission. *Neuron* **105**, 678–687.e5 (2020) Publisher: Elsevier.
60. PR Territo, SA French, MC Dunleavy, FJ Evans, RS Balaban, Calcium activation of heart mitochondrial oxidative phosphorylation. Rapid kinetics of mV O2 , NADH, and light scattering. *J. Biol. Chem.* **276**, 2586–2599 (2001).
61. AL Hodgkin, AF Huxley, A quantitative description of membrane current and its application to conduction and excitation in nerve. *The J. Physiol.* **117**, 500–544 (1952).
62. JS Rothman, PB Manis, The Roles Potassium Currents Play in Regulating the Electrical Activity of Ventral Cochlear Nucleus Neurons. *J. Neurophysiol.* **89**, 3097–3113 (2003) Publisher: American Physiological Society.
63. TP Vogels, LF Abbott, Signal Propagation and Logic Gating in Networks of Integrate-and-Fire Neurons. *J. Neurosci.* **25**, 10786–10795 (2005) Publisher: Society for Neuroscience Section: Behavioral/Systems/Cognitive.
64. G Van Rossum, FL Drake, *Python 3 Reference Manual.* (CreateSpace, Scotts Valley, CA), (2009).
65. CR Harris, et al., Array programming with NumPy. *Nature* **585**, 357–362 (2020) Bandiera_abtest: a Cc_license_type: cc_by Cg_type: Nature Research Journals Number: 7825 Primary_atype: Reviews Publisher: Nature Publishing Group Subject_term: Computational neuroscience;Computational science;Computer science;Software;Solar physics Subject_term_id: computational-neuroscience;computational-science;computer-science;software;solar-physics.
66. M Stimberg, R Brette, DF Goodman, Brian 2, an intuitive and efficient neural simulator. *eLife* **8**, e47314 (2019) Publisher: eLife Sciences Publications, Ltd.
67. J Alstott, E Bullmore, D Plenz, powerlaw: A Python Package for Analysis of Heavy-Tailed Distributions. *PLOS ONE* **9**, e85777 (2014) Publisher: Public Library of Science.
68. GA Ascoli, DE Donohue, M Halavi, NeuroMorpho.Org: A Central Resource for Neuronal Morphologies. *J. Neurosci.* **27**, 9247–9251 (2007) ISBN: 2712005775.
69. JD Hunter, Matplotlib: A 2D Graphics Environment. *Comput. Sci. Eng.* **9**, 90–95 (2007) Conference Name: Computing in Science Engineering.
70. N Sada, S Lee, T Katsu, T Otsuki, T Inoue, Targeting LDH enzymes with a stiripentol analog to treat epilepsy. *Science* **347**, 1362–1367 (2015) Publisher: American Association for the Advancement of Science Section: Report.
71. FR Fernandez, B Rahsepar, JA White, Differences in the Electrophysiological Properties of Mouse Somatosensory Layer 2/3 Neurons In Vivo and Slice Stem from Intrinsic Sources Rather than a Network-Generated High Conductance State. *eNeuro* **5** (2018) Publisher: Society for Neuroscience.
72. AB Nelson, CM Krispel, C Sekirnjak, SD Lac, Long-lasting increases in intrinsic excitability triggered by inhibition. *Neuron* **40**, 609–620 (2003).
73. GS Salomons, et al., X-linked creatine transporter defect: an overview. *J. Inherit. Metab. Dis.* **26**, 309–318 (2003).
74. I D'Andrea Meira, et al., Ketogenic Diet and Epilepsy: What We Know So Far. *Front. Neurosci.* **13**, 5 (2019).
75. CJ Barborka, KETOGENIC DIET TREATMENT OF EPILEPSY IN ADULTS. *J. Am. Med. Assoc.* **91**, 73 (1928).
76. AKM Hammarström, PW Gage, Inhibition of oxidative metabolism increases persistent sodium current in rat CA1 hippocampal neurons. *The J. Physiol.* **510**, 735–741 (1998).
77. AK Hammarström, PW Gage, Hypoxia and persistent sodium current. *Eur. Biophys. J.* **31**, 323–330 (2002).
78. Jh Ma, At Luo, Ph Zhang, Effect of hydrogen peroxide on persistent sodium current in guinea pig ventricular myocytes. *Acta Pharmacol. Sinica* **26**, 828–834 (2005) Number: 7 Publisher: Nature Publishing Group.
79. A Luo, J Ma, P Zhang, H Zhou, W Wang, Sodium channel gating modes during redox reaction. *Cell. Physiol. Biochem.* **19**, 9–20 (2007) ISBN: 8627688621.
80. Y Qu, et al., Differential Modulation of Sodium Channel Gating and Persistent Sodium Currents by the 1, 2, and 3 Subunits. *Mol. Cell. Neurosci.* **18**, 570–580 (2001).
81. TK Aman, et al., Regulation of persistent na current by interactions between subunits of voltage-gated na channels. *J. Neurosci.* **29**, 2027–2042 (2009).
82. YY Vilin, CH Peters, PC Ruben, Acidosis Differentially Modulates Inactivation in NaV1.2, NaV1.4, and NaV1.5 Channels. *Front. Pharmacol.* **3** (2012).
83. MR Ghovanloo, CH Peters, PC Ruben, Effects of acidosis on neuronal voltage-gated sodium channels: Nav1.1 and Nav1.3. *Channels* **12**, 367–377 (2018).
84. AR Cantrell, WA Catterall, Neuromodulation of Na + channels: An unexpected form of cellular platicity. *Nat. Rev. Neurosci.* **2**, 397–407 (2001) Bandiera_abtest: a Cg_type: Nature Research Journals Number: 6 Primary_atype: Reviews Publisher: Nature Publishing Group.
85. M Kassmann, et al., Oxidation of multiple methionine residues impairs rapid sodium channel inactivation. *Pflugers Arch. Eur. J. Physiol.* **456**, 1085–1095 (2008).
86. S Kaneko, et al., A critical role of TRPM2 in neuronal cell death by hydrogen peroxide. *J. Pharmacol. Sci.* **101**, 66–76 (2006).
87. AL Perraud, et al., Accumulation of free ADP-ribose from mitochondria mediates oxidative stress-induced gating of TRPM2 cation channels. *The J. Biol. Chem.* **280**, 6138–6148 (2005).
88. WR Evans, A San Pietro, Phosphorolysis of adenosine diphosphoribose. *Arch. Biochem. Biophys.* **113**, 236–244 (1966).

89. M Kolisek, A Beck, A Fleig, R Penner, Cyclic ADP-Ribose and Hydrogen Peroxide Synergize with ADP-Ribose in the Activation of TRPM2 Channels. *Mol. Cell* **18**, 61–69 (2005).
90. N Byczkowicz, et al., HCN channel-mediated neuromodulation can control action potential velocity and fidelity in central axons. *eLife* **8**, e42766 (2019).
91. M Biel, C Wahl-Schott, S Michalakis, X Zong, Hyperpolarization-Activated Cation Channels: From Genes to Function. *Physiol. Rev.* **89**, 847–885 (2009).
92. JP Ruppersberg, et al., Regulation of fast inactivation of cloned mammalian I K (A) channels by cysteine oxidation. *Nature* **352**, 711–714 (1991) Bandiera_abtest: a Cg_type: Nature Research Journals Number: 6337 Primary_atype: Research Publisher: Nature Publishing Group.
93. J Weng, Y Cao, N Moss, M Zhou, Modulation of voltage-dependent shaker family potassium channels by an aldo-keto reductase. *J. Biol. Chem.* **281**, 15194–15200 (2006).
94. D Oliver, et al., Functional Conversion Between A-Type and Delayed Rectifier K+ Channels by Membrane Lipids. *Science* **304**, 265–270 (2004) Publisher: American Association for the Advancement of Science Section: Research Article.
95. N Gu, K Vervaeke, JF Storm, BK potassium channels facilitate high-frequency firing and cause early spike frequency adaptation in rat CA1 hippocampal pyramidal cells. *J. Physiol.* **580**, 859–882 (2007).
96. TJ DiChiara, PH Reinhart, Redox Modulation of hslo Ca2+-Activated K+ Channels. *The J. Neurosci.* **17**, 4942–4955 (1997).
97. H Soh, W Jung, DY Uhm, S Chung, Modulation of large conductance calcium-activated potassium channels from rat hippocampal neurons by glutathione. *Neurosci. Lett.* **298**, 115–118 (2001).
98. T Vaithianathan, et al., Direct regulation of BK channels by phosphatidylinositol 4,5-bisphosphate as a novel signaling pathway. *The J. Gen. Physiol.* **132**, 13–28 (2008).
99. QY Tang, Z Zhang, XY Meng, M Cui, DE Logothetis, Structural Determinants of Phosphatidylinositol 4,5-Bisphosphate (PIP2) Regulation of BK Channel Activity through the RCK1 Ca2+ Coordination Site*. *J. Biol. Chem.* **289**, 18860–18872 (2014).
100. PM Joksovic, et al., CaV3.2 is the major molecular substrate for redox regulation of T-type Ca2+ channels in the rat and mouse thalamus. *J. Physiol.* **574**, 415–430 (2006).
101. SM Todorovic, et al., Redox modulation of T-Type calcium channels in rat peripheral nociceptors. *Neuron* **31**, 75–85 (2001).
102. JA Kim, et al., Augmentation of Cav3.2 T-Type Calcium Channel Activity by cAMP-Dependent Protein Kinase A. *J. Pharmacol. Exp. Ther.* **318**, 230–237 (2006).
103. TM Grieco, FS Afshari, IM Raman, A Role for Phosphorylation in the Maintenance of Resurgent Sodium Current in Cerebellar Purkinje Neurons. *J. Neurosci.* **22**, 3100–3107 (2002) Publisher: Society for Neuroscience Section: ARTICLE.
104. TM Grieco, JD Malhotra, C Chen, LL Isom, IM Raman, Open-channel block by the cytoplasmic tail of sodium channel beta4 as a mechanism for resurgent sodium current. *Neuron* **45**, 233–244 (2005).
105. AH Lewis, IM Raman, Resurgent current of voltage-gated Na $^+$ channels. *The J. Physiol.* **592**, 4825–4838 (2014).
106. A Chu, E Stefani, Phosphatidylinositol 4,5-bisphosphate-induced Ca2+ release from skeletal muscle sarcoplasmic reticulum terminal cisternal membranes. Ca2+ flux and single channel studies. *The J. Biol. Chem.* **266**, 7699–7705 (1991).
107. Z He, S Feng, Q Tong, DW Hilgemann, KD Philipson, Interaction of PIP2 with the XIP region of the cardiac Na/Ca exchanger. *Am. J. Physiol. Physiol.* **278**, C661–C666 (2000) Publisher: American Physiological Society.
108. MV Avshalumov, BT Chen, T Koos, JM Tepper, ME Rice, Endogenous hydrogen peroxide regulates the excitability of midbrain dopamine neurons via ATP-sensitive potassium channels. *J. Neurosci.* **25**, 4222–4231 (2005).
109. CT Bond, J Maylie, JP Adelman, SK channels in excitability, pacemaking and synaptic integration. *Curr. Opin. Neurobiol.* **15**, 305–311 (2005).
110. I Lange, R Penner, A Fleig, A Beck, Synergistic regulation of endogenous TRPM2 channels by adenine dinucleotides in primary human neutrophils. *Cell Calcium* **44**, 604–615 (2008).
111. K Venkatachalam, F Zheng, DL Gill, Regulation of Canonical Transient Receptor Potential (TRPC) Channel Function by Diacylglycerol and Protein Kinase C*. *J. Biol. Chem.* **278**, 29031–29040 (2003).
112. A Herrero-Mendez, et al., The bioenergetic and antioxidant status of neurons is controlled by continuous degradation of a key glycolytic enzyme by APC/C–Cdh1. *Nat. Cell Biol.* **11**, 747–752 (2009) Number: 6 Publisher: Nature Publishing Group.
113. E Suberbielle, et al., Physiologic brain activity causes DNA double-strand breaks in neurons, with exacerbation by amyloid-. *Nat. Neurosci.* **16**, 613–621 (2013) Number: 5 Publisher: Nature Publishing Group.
114. PJ Magistretti, O Sorg, JL Martin, CHAPTER 11 - Regulation of Glycogen Metabolism in Astrocytes: Physiological, Pharmacological, and Pathological Aspects in *Astrocytes*, ed. S Murphy. (Academic Press, Boston), pp. 243–265 (1993).
115. AM Brown, Brain glycogen re-awakened. *J. Neurochem.* **89**, 537–552 (2004) _eprint: https://onlinelibrary.wiley.com/doi/pdf/10.1111/j.1471-4159.2004.02421.x.
116. MJ Gaudry, M Jastroch, Molecular evolution of uncoupling proteins and implications for brain function. *Neurosci. Lett.* **696**, 140–145 (2019).
117. HP Lambert, et al., Control of Mitochondrial pH by Uncoupling Protein 4 in Astrocytes Promotes Neuronal Survival *. *J. Biol. Chem.* **289**, 31014–31028 (2014) Publisher: Elsevier.
118. S Diano, et al., Uncoupling Protein 2 Prevents Neuronal Death Including that Occurring during Seizures: A Mechanism

for Preconditioning. *Endocrinology* **144**, 5014–5021 (2003).
119. O Braissant, H Henry, E Béard, J Uldry, Creatine deficiency syndromes and the importance of creatine synthesis in the brain. *Amino Acids* **40**, 1315–1324 (2011).
120. Q Song, B Meng, H Xu, Z Mao, The emerging roles of vacuolar-type ATPase-dependent Lysosomal acidification in neurodegenerative diseases. *Transl. Neurodegener.* **9**, 17 (2020).
121. C Pulido, TA Ryan, Synaptic vesicle pools are a major hidden resting metabolic burden of nerve terminals. *Sci. Adv.* **7**, eabi9027 (2021) Publisher: American Association for the Advancement of Science.
122. NJ Pavlos, R Jahn, Distinct yet overlapping roles of Rab GTPases on synaptic vesicles. *Small GTPases* **2**, 77–81 (2011).
123. P Mishra, DC Chan, Metabolic regulation of mitochondrial dynamics. *The J. Cell Biol.* **212**, 379–387 (2016).
124. RJ Youle, AM van der Bliek, Mitochondrial Fission, Fusion, and Stress. *Sci. (New York, N.Y.)* **337**, 1062–1065 (2012).
125. L Guillaud, SE El-Agamy, M Otsuki, M Terenzio, Anterograde Axonal Transport in Neuronal Homeostasis and Disease. *Front. Mol. Neurosci.* **13** (2020).
126. D Nelson, M Cox, *Lehninger Principles of Biochemistry.* (Worth publishers, New York), 3 edition, (2000).
127. B Alberts, et al., *Essential cell biology.* (W. W. Norton & Company), 5 edition, (2019).
128. RA Swanson, MM Morton, SM Sagar, FR Sharp, Sensory stimulation induces local cerebral glycogenolysis: demonstration by autoradiography. *Neuroscience* **51**, 451–461 (1992).
129. GA Dienel, KK Ball, NF Cruz, A glycogen phosphorylase inhibitor selectively enhances local rates of glucose utilization in brain during sensory stimulation of conscious rats: implications for glycogen turnover. *J. Neurochem.* **102**, 466–478 (2007).
130. MS Ioannou, et al., Neuron-Astrocyte Metabolic Coupling Protects against Activity-Induced Fatty Acid Toxicity. *Cell* **177**, 1522–1535.e14 (2019).
